# Correction: Self-allocation bias in performance-based cooperative decisions is driven by self-interest rather than distorted performance encoding

**DOI:** 10.1371/journal.pbio.3003967

**Published:** 2026-08-21

**Authors:** Sihui Zhang, Xue Yong, Yina Ma, Christoph W. Korn

In Fig 3, the color labeling in panel A and the corresponding description in the figure caption were incorrect. The authors have provided a corrected Fig 3 and updated figure legend below.

**Fig 3:**
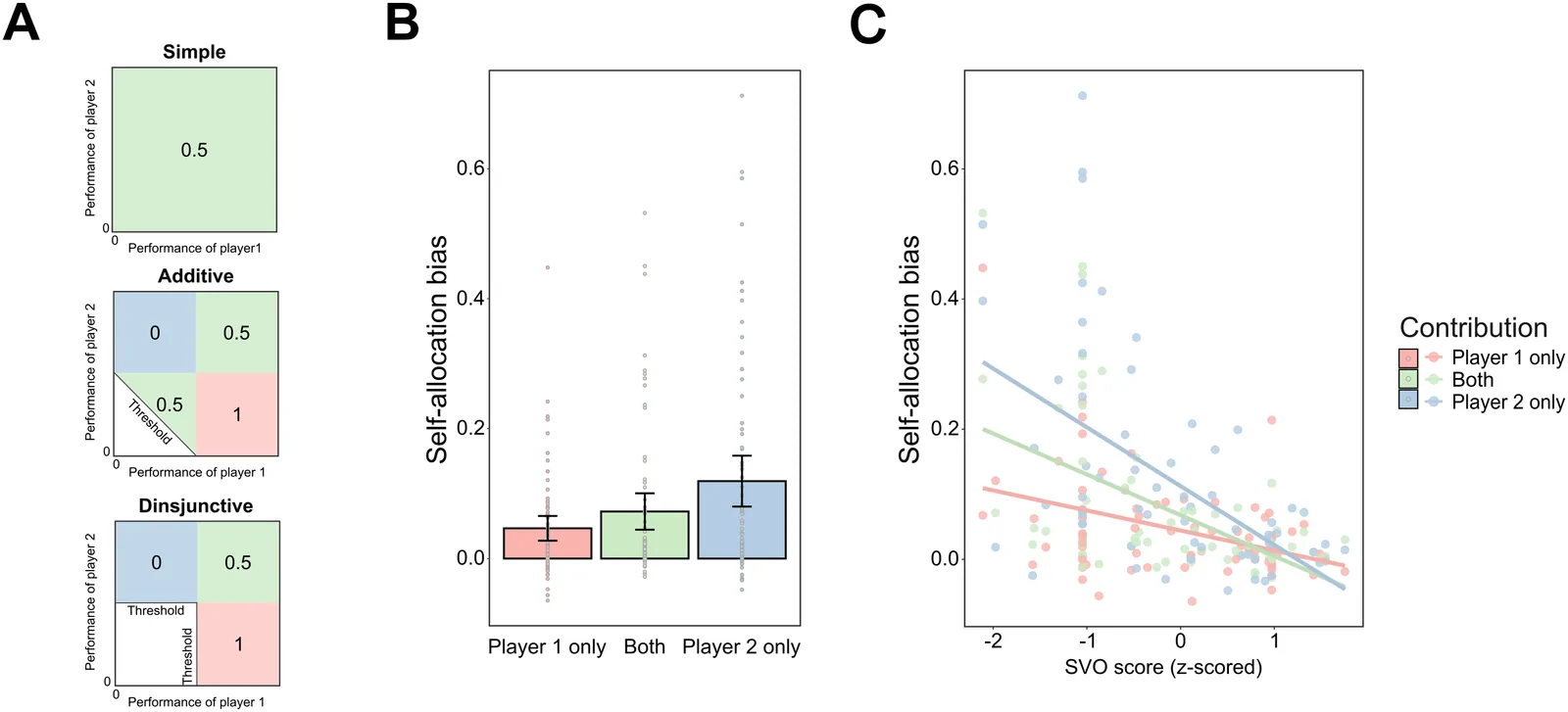
Contribution categorization and its effect on self-allocation bias. **(A)** The categorization of contributions with respect to Shapley values determines “optimal” reward allocation according to individual contribution based on collective task criteria. Rewards are distributed exclusively in the “colored” areas. The values shown within colored squares denote the allocation ratios assigned to player 1, as calculated by the Shapley value. Contribution structure is classified into three categories: a value of 1 indicates rewards are allocated entirely to player 1 (“player1 only”, red area); a value of 0.5 indicates rewards are shared equally between two players (“both”, green area); and a value of 0 indicates rewards are allocated entirely to player 2 (“player2 only”, blue area). **(B)** The self-allocation bias was larger in the “player2 only” condition than in the “both” and the “player1 only” conditions. The self-allocation bias was calculated as the relative allocation difference between self-relevant and self-irrelevant conditions. That is, the bias was highest when participants’ performance did not contribute to the joint outcome. **(C)** In all contribution structures, SVO scores were negatively associated with the self-allocation bias, showing that participants with lower SVO scores had a larger self-bias. The slopes decreased from “player1 only” condition to the “both” condition, and then to the “player2 only” condition. Note: Panels (B) and (C) display the self-allocation bias averaged across trials within each participant for visualization only; all statistical inferences were derived from hierarchical Bayesian multilevel models applied to the full trial-level data. The data and code for panels (B) and (C) are available at https://doi.org/10.17605/OSF.IO/AFDQS.

## References

[1] Zhang S, Yong X, Ma Y, Korn CW. Self-allocation bias in performance-based cooperative decisions is driven by self-interest rather than distorted performance encoding. PLoS Biol. 2026;24(3):e3003694. doi: 10.1371/journal.pbio.3003694 41886463 PMC13020808

