# Supplementary figures and images for: Self-allocation bias in performance-based cooperative decisions is driven by self-interest rather than distorted performance encoding
Source: PLoS Biol. 2026 Mar 26;24(3):e3003694. doi: 10.1371/journal.pbio.3003694 (PMC13020808; doi:10.1371/journal.pbio.3003694)

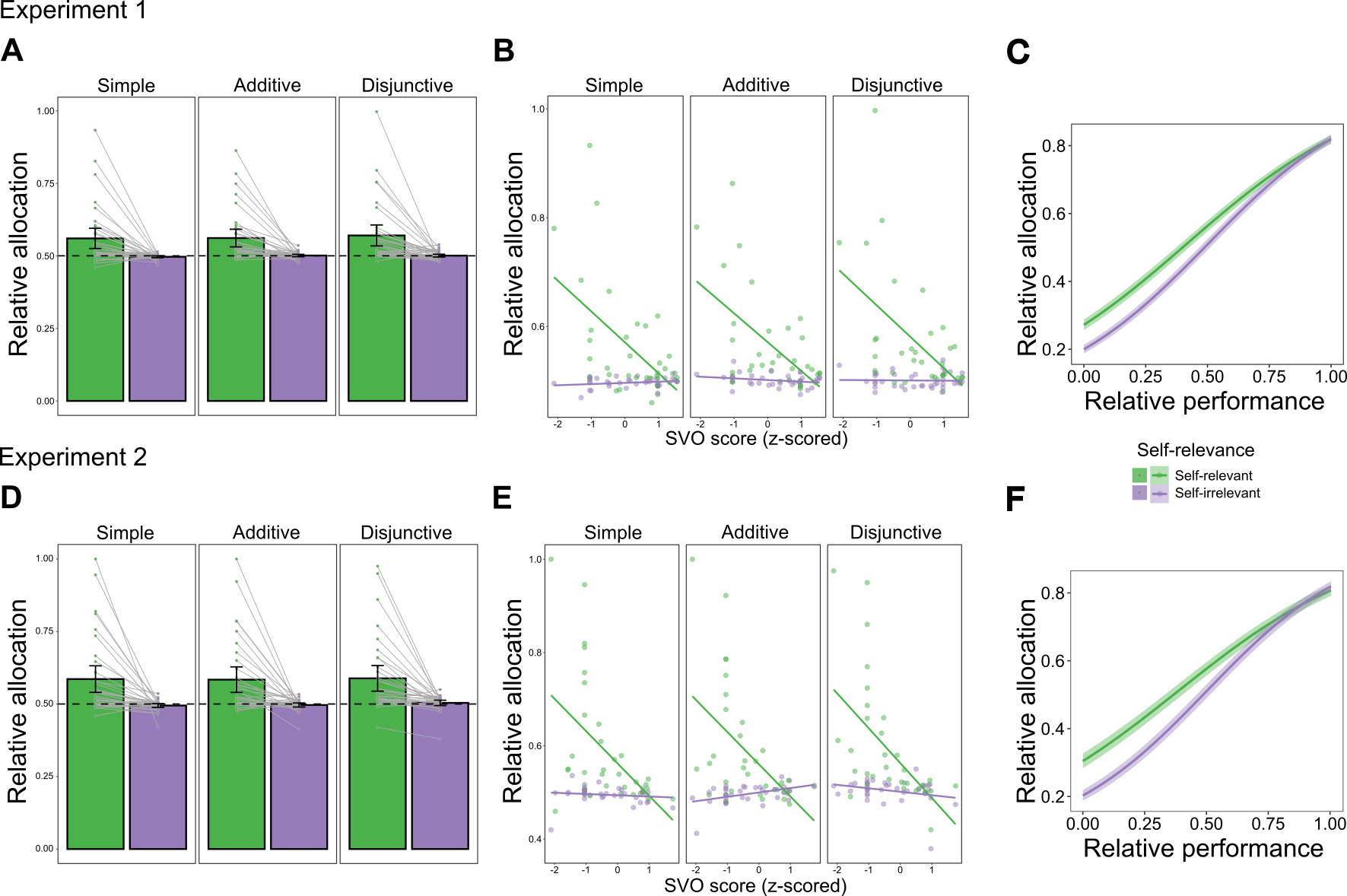

Supplement: S1 Fig — Panels (A–C) present results from Experiment 1, and panels (D–F) present results from Experiment 2. All statistical analyses were conducted using hierarchical Bayesian multilevel models, and the results remained robust across analyses. The data and code used to generate this figure are available at https://doi.org/10.17605/OSF.IO/AFDQS. (TIFF) [file pbio.3003694.s001.tiff]

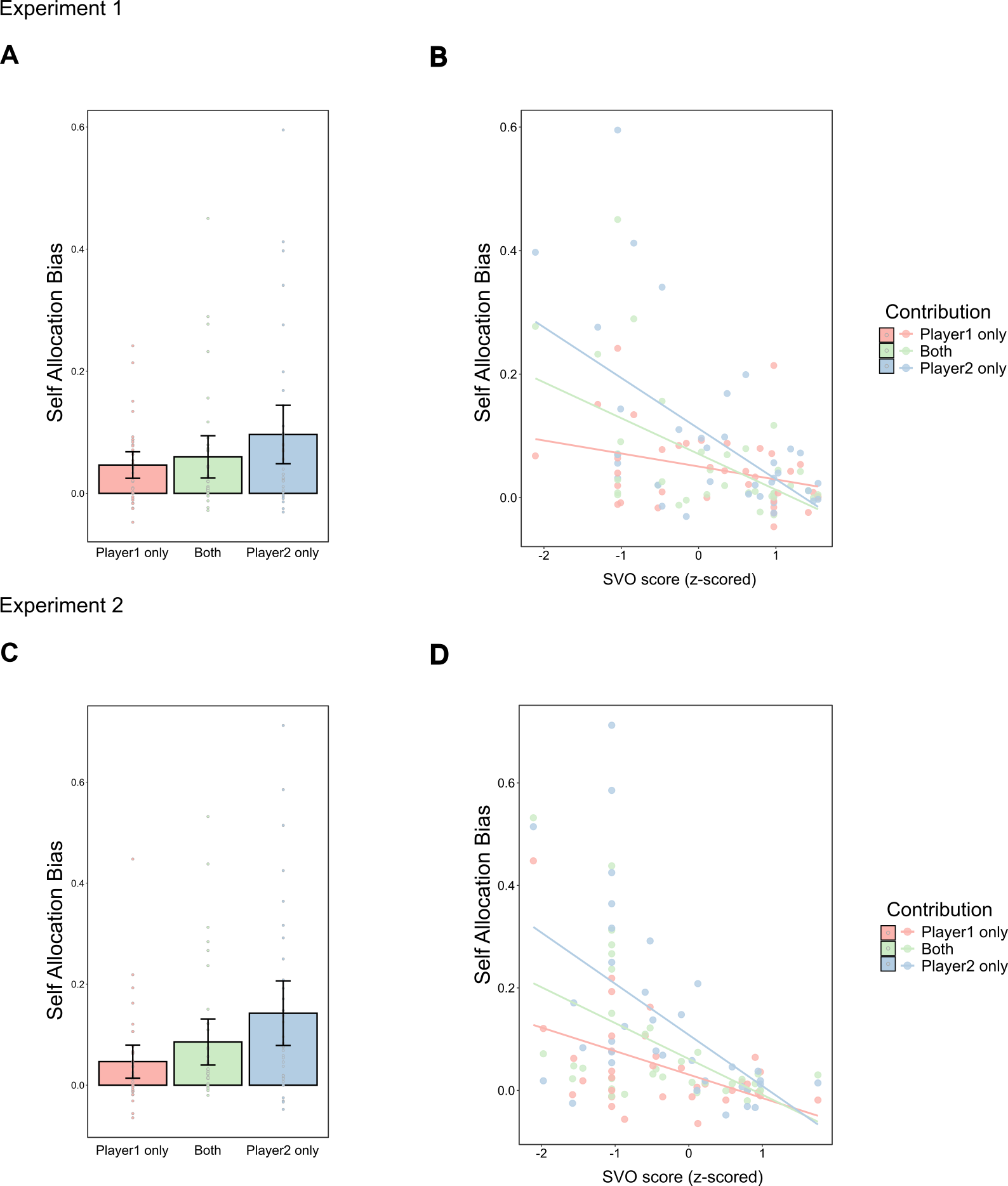

Supplement: S2 Fig — Panels (A, B) show results from Experiment 1, and panels (C, D) show results from Experiment 2. All statistical tests were conducted using hierarchical Bayesian multilevel models and the results remained robust. The data and code used to generate this figure are available at https://doi.org/10.17605/OSF.IO/AFDQS. (TIFF) [file pbio.3003694.s002.tiff]

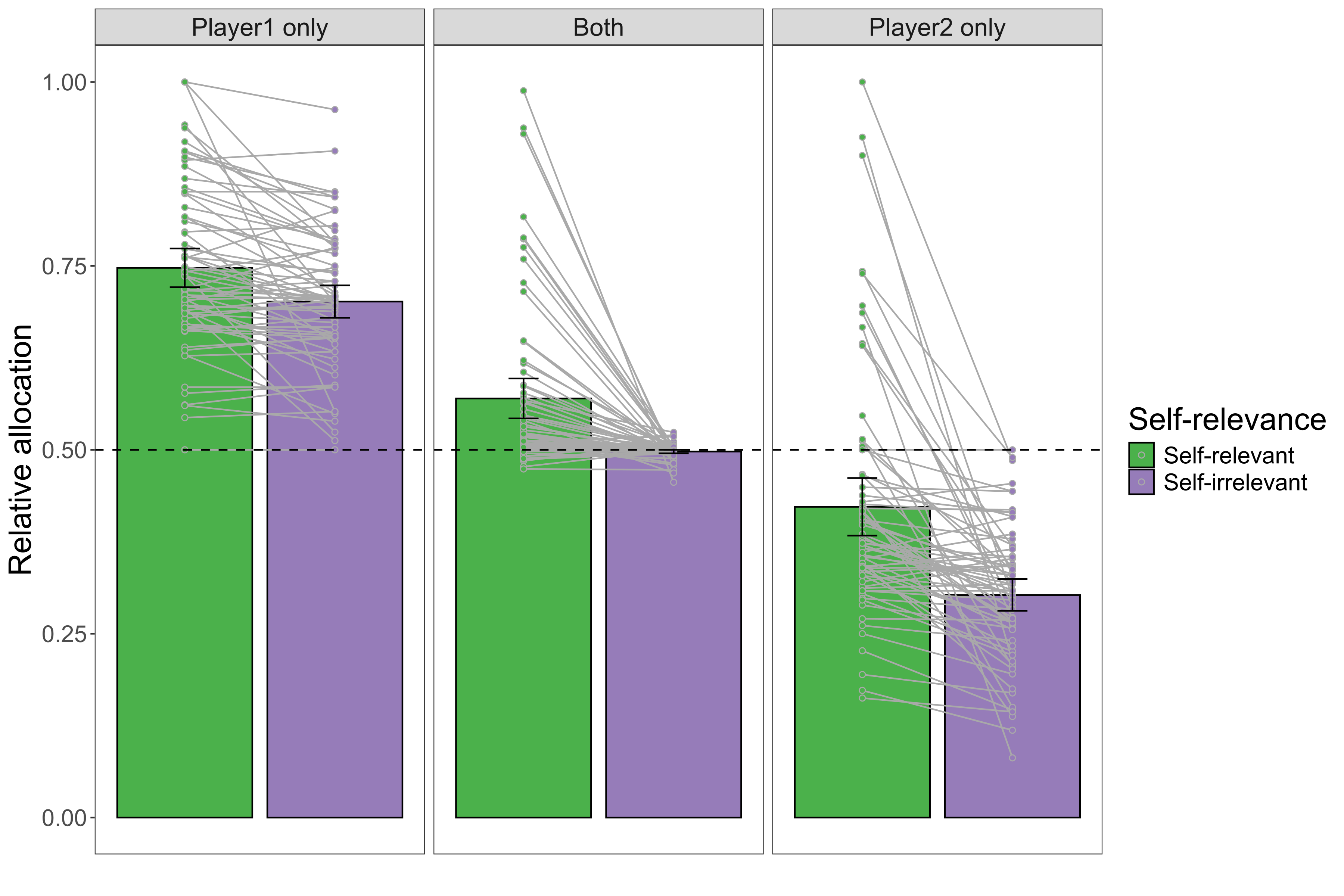

Supplement: S3 Fig — The data and code used to generate this figure are available at https://doi.org/10.17605/OSF.IO/AFDQS. (TIFF) [file pbio.3003694.s003.tiff]

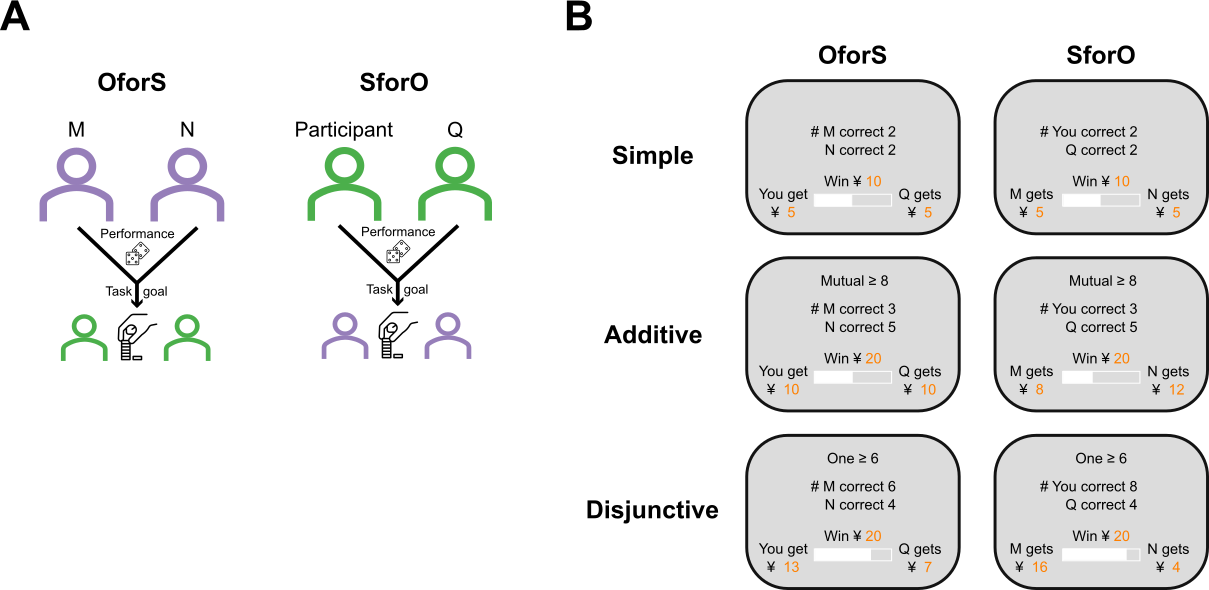

Supplement: S4 Fig — (A) Conceptual diagram of the two additional conditions introduced in Experiment 3. The SforS (self-performance for self-allocation) and OforO (other-performance for other-allocation) conditions correspond to the self-relevant and self-irrelevant conditions in Experiments 1 and 2, respectively, where self-relevance in both performance source and allocation recipient is consistent. The two additional conditions: SforO (self-performance for other-allocation) and OforS (other-performance for self-allocation), introduce crossed combinations of self-relevance between performance source and allocation recipient. (B) Example task frames for the OforS and SforO conditions in Experiment 3. (TIFF) [file pbio.3003694.s004.tiff]

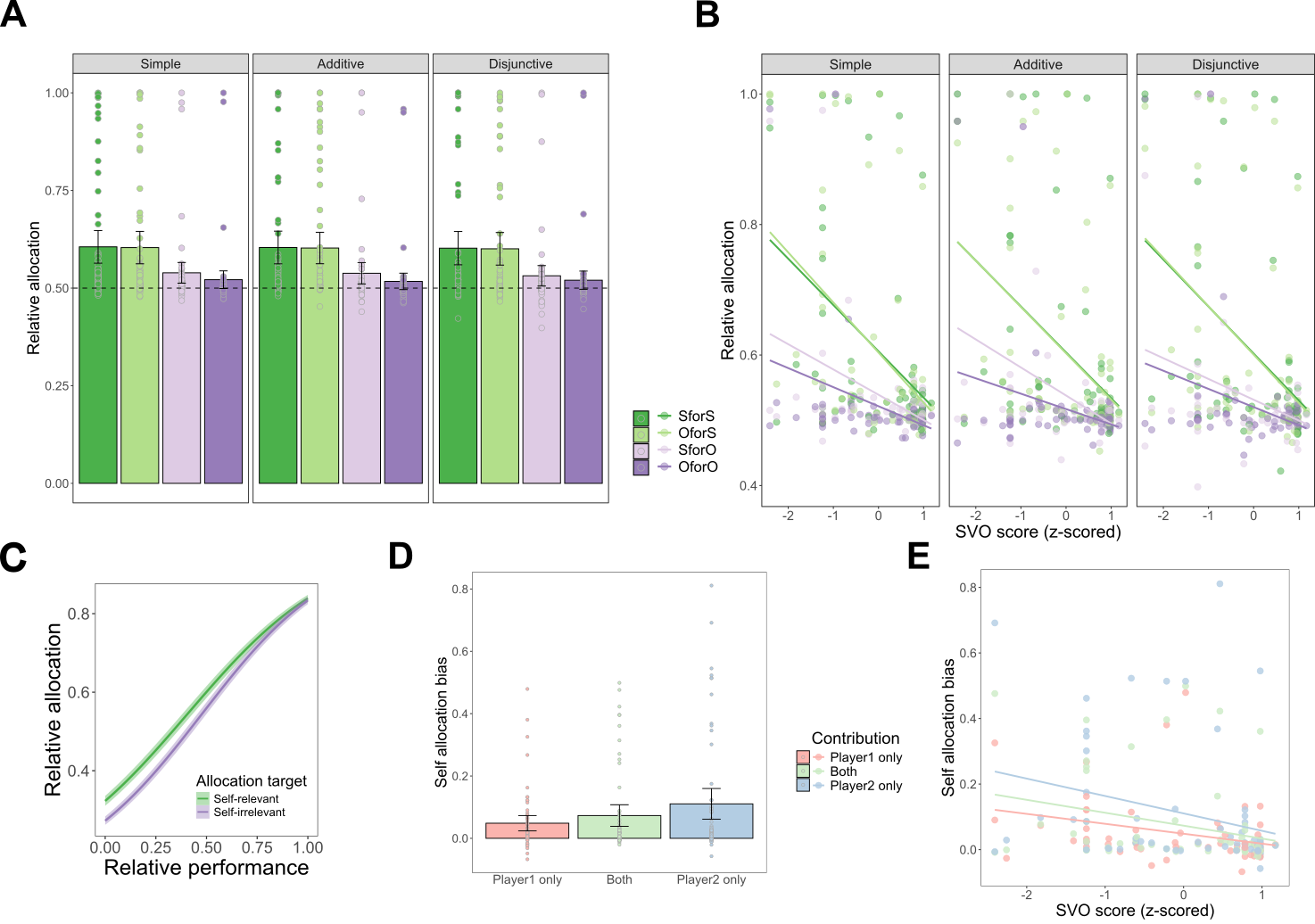

Supplement: S5 Fig — (A) Relative allocations across all conditions. (B) Associations between SVO scores and relative allocations. (C) Slopes of relative performance predicting relative allocation. (D) Self-allocation bias across three contribution structures. (E) Associations between self-allocation bias and SVO scores. For illustration, data from the four conditions are plotted separately. In the actual analysis, these four conditions were modeled using two factors (allocation target and performance source) within hierarchical Bayesian multilevel models. The effect of allocation target was consistent with the self-relevance effect observed in Experiments 1 and 2. The data and code used to generate this figure are available at https://doi.org/10.17605/OSF.IO/AFDQS. (TIFF) [file pbio.3003694.s005.tiff]

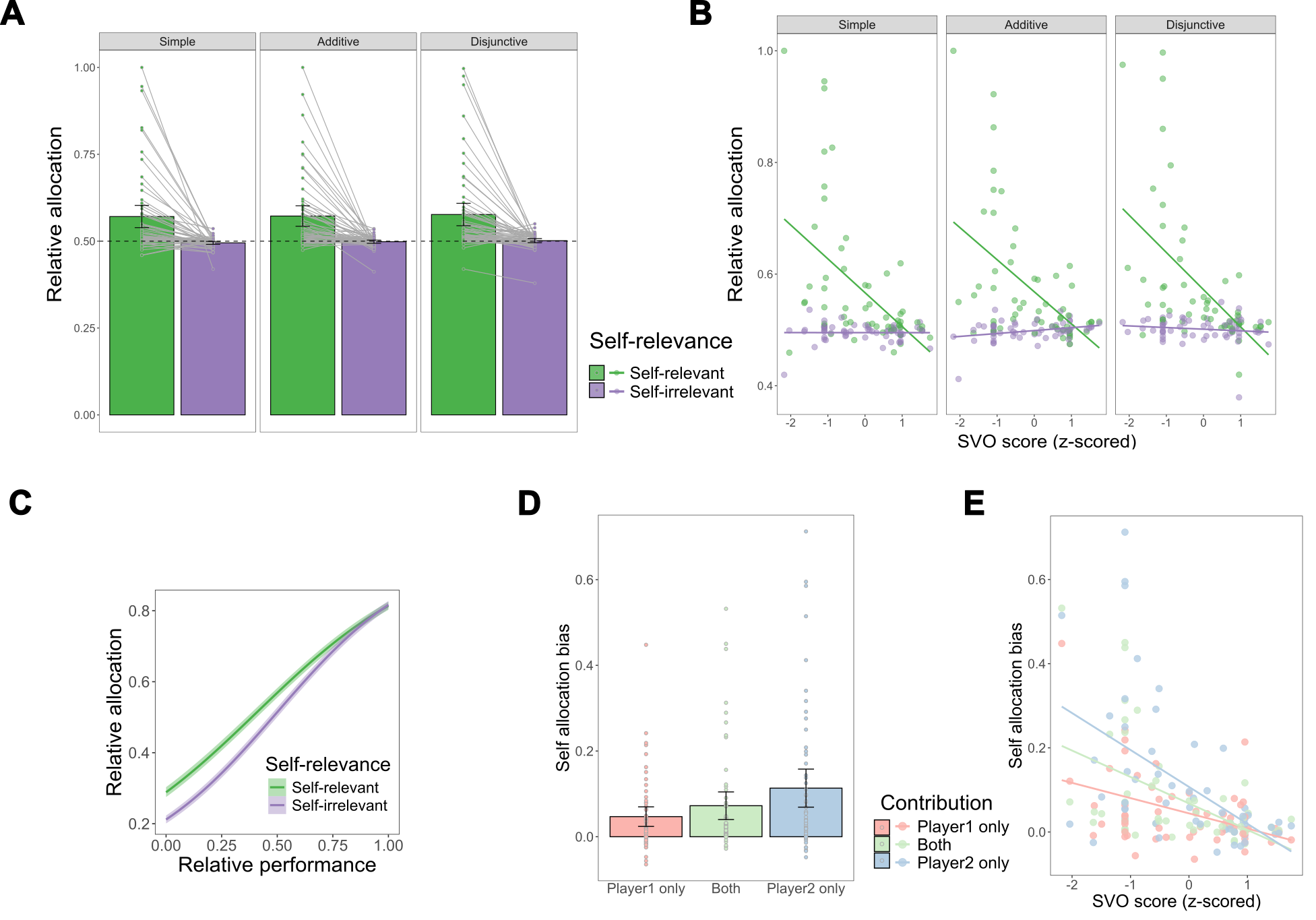

Supplement: S6 Fig — (A) Relative allocations across all conditions. (B) Associations between SVO scores and relative allocations. (C) Slopes of relative performance predicting relative allocation. (D) Self-allocation bias across three contribution structures. (E) Associations between self-allocation bias and SVO scores. All statistical tests were conducted using hierarchical Bayesian multilevel models and the results remained robust after exclusion. The data and code used to generate this figure are available at https://doi.org/10.17605/OSF.IO/AFDQS. (TIFF) [file pbio.3003694.s006.tiff]

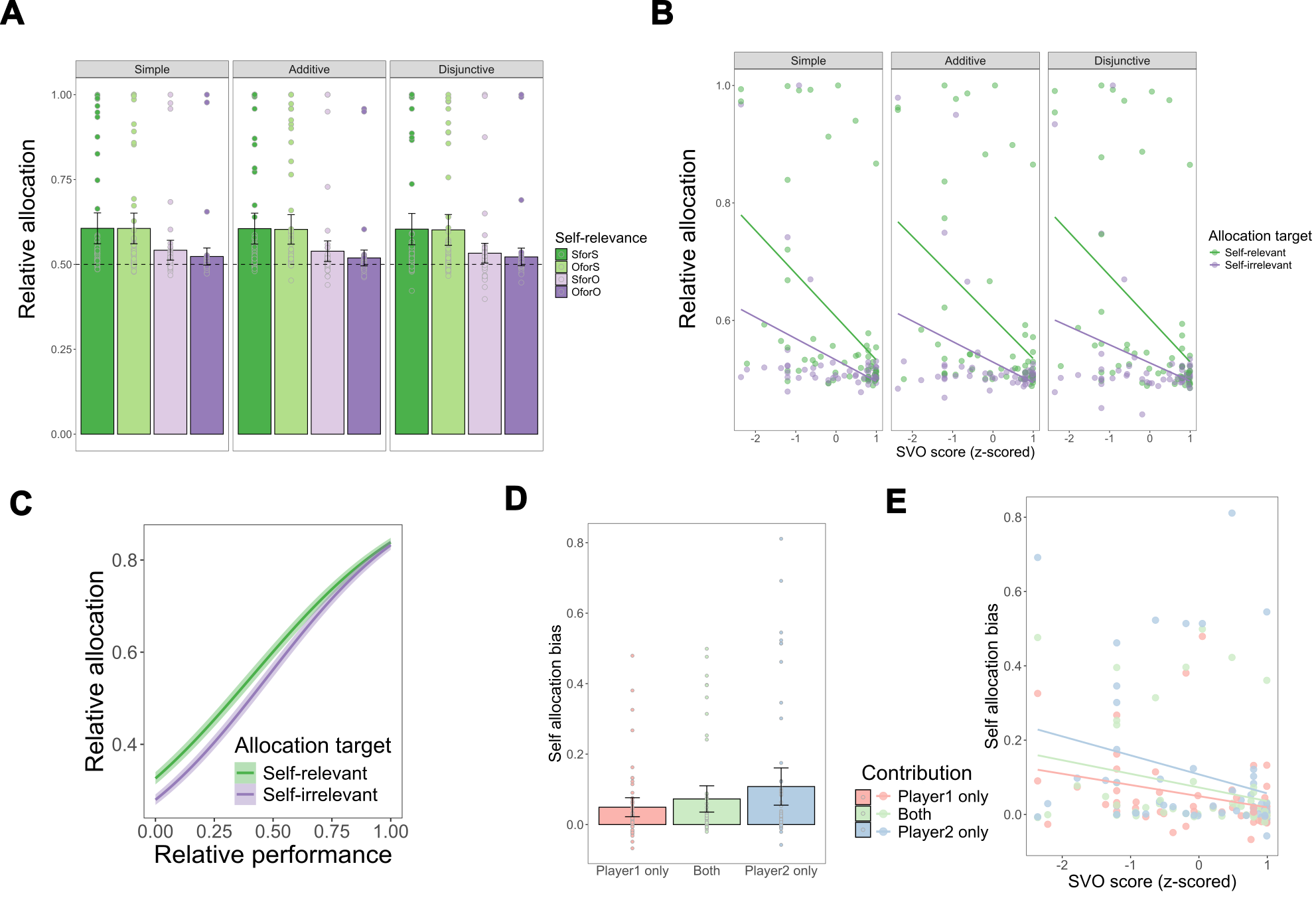

Supplement: S7 Fig — (A) Relative allocations across all conditions. (B) Associations between SVO scores and relative allocations. (C) Slopes of relative performance predicting relative allocation. (D) Self-allocation bias across three contribution structures. (E) Associations between self-allocation bias and SVO scores. All statistical tests were conducted using hierarchical Bayesian multilevel models and the results remained robust after exclusion. The data and code used to generate this figure are available at https://doi.org/10.17605/OSF.IO/AFDQS. (TIFF) [file pbio.3003694.s007.tiff]
