# Supplementary material for: Self-allocation bias in performance-based cooperative decisions is driven by self-interest rather than distorted performance encoding
Source: PLoS Biol. 2026 Mar 26;24(3):e3003694. doi: 10.1371/journal.pbio.3003694 (PMC13020808; doi:10.1371/journal.pbio.3003694)
Supplement: S1 Appendix — (DOCX) [file pbio.3003694.s008.docx]

# **S1 Appendix**

All statistical analysis related to the model (*Formular 1*) testing the effect of self-relevance and collective task criteria on relative allocation in the combined sample, or Experiment 1 and 2 respectively.

*Combined experiments*

**Table A.** The effect of self-relevance and collective task criteria on relative allocation.

|  | Estimate | Est.Error | l-95% CI | u-95% CI | Rhat | Bulk_ESS | Tail_ESS |
| --- | --- | --- | --- | --- | --- | --- | --- |
| Intercept | 0.19 | 0.03 | 0.14 | 0.24 | 1 | 7442.84 | 10353.01 |
| taskAdditive | 0.03 | 0.03 | -0.03 | 0.08 | 1 | 15474.54 | 13442.32 |
| taskDisjunctive | 0.02 | 0.03 | -0.04 | 0.07 | 1 | 15633.65 | 12552.93 |
| conditionSelf-irrelevant | -0.19 | 0.03 | -0.24 | -0.14 | 1 | 13396.59 | 13076.25 |

**Table B.** Post-hoc pairwise tests for self-relevant vs. self-irrelevant conditions on relative allocation.

| Task | odds.ratio | lower.HPD | upper.HPD |
| --- | --- | --- | --- |
| Simple | 1.21 | 1.15 | 1.28 |
| Additive | 1.25 | 1.19 | 1.32 |
| Disjunctive | 1.23 | 1.17 | 1.30 |

*Note: Contrast: (Self-relevant) / (Self-irrelevant)*

**Table C.** Posterior estimates for relative allocation in all experimental conditions.

| Task | Self-relevance | response | lower.HPD | upper.HPD |
| --- | --- | --- | --- | --- |
| Simple | Self-relevant | 0.55 | 0.53 | 0.56 |
| Additive | Self-relevant | 0.55 | 0.54 | 0.57 |
| Disjunctive | Self-relevant | 0.55 | 0.54 | 0.56 |
| Simple | Self-irrelevant | 0.50 | 0.49 | 0.51 |
| Additive | Self-irrelevant | 0.50 | 0.49 | 0.51 |
| Disjunctive | Self-irrelevant | 0.50 | 0.49 | 0.51 |

*Experiment 1*

**Table D.** The effect of self-relevance and collective task criteria on relative allocation in Exp 1.

| Intercept | 0.17 | 0.03 | 0.1 | 0.23 | 1 | 5884.16 | 9389.25 |
| --- | --- | --- | --- | --- | --- | --- | --- |
| taskAdditive | 0.04 | 0.03 | -0.02 | 0.11 | 1 | 13117.28 | 13364.31 |
| taskDisjunctive | 0.03 | 0.03 | -0.03 | 0.1 | 1 | 13289.21 | 13472.02 |
| conditionSelf-irrelevant | -0.17 | 0.03 | -0.23 | -0.1 | 1 | 11159.23 | 12972.53 |

**Table E.** Post-hoc pairwise tests for self-relevant vs. self-irrelevant conditions on relative allocation in Exp 1.

| Task | odds.ratio | lower.HPD | upper.HPD |
| --- | --- | --- | --- |
| Simple | 1.18 | 1.10 | 1.26 |
| Additive | 1.23 | 1.15 | 1.31 |
| Disjunctive | 1.22 | 1.14 | 1.30 |

*Note: Contrast: (Self-relevant) / (Self-irrelevant)*

**Table F.** Posterior estimates for relative allocation in all experimental conditions in Exp 1.

| Task | Self-relevance | response | lower.HPD | upper.HPD |
| --- | --- | --- | --- | --- |
| Simple | Self-relevant | 0.54 | 0.52 | 0.56 |
| Additive | Self-relevant | 0.55 | 0.54 | 0.57 |
| Disjunctive | Self-relevant | 0.55 | 0.53 | 0.57 |
| Simple | Self-irrelevant | 0.50 | 0.48 | 0.52 |
| Additive | Self-irrelevant | 0.50 | 0.48 | 0.52 |
| Disjunctive | Self-irrelevant | 0.50 | 0.48 | 0.52 |

*Experiment 2*

**Table G.** The effect of self-relevance and collective task criteria on relative allocation in Exp 2.

|  | Estimate | Est.Error | l-95% CI | u-95% CI | Rhat | Bulk_ESS | Tail_ESS |
| --- | --- | --- | --- | --- | --- | --- | --- |
| Intercept | 0.23 | 0.04 | 0.15 | 0.32 | 1 | 8428.25 | 10274.21 |
| taskAdditive | 0.01 | 0.05 | -0.08 | 0.1 | 1 | 14474.14 | 12797.36 |
| taskDisjunctive | -0.01 | 0.05 | -0.1 | 0.09 | 1 | 14688.53 | 13739.61 |
| conditionSelf-irrelevant | -0.24 | 0.05 | -0.33 | -0.14 | 1 | 12754.83 | 12991.39 |

**Table H.** Post-hoc pairwise tests for self-relevant vs. self-irrelevant conditions on relative allocation in Exp 2.

| Task | odds.ratio | lower.HPD | upper.HPD |
| --- | --- | --- | --- |
| Simple | 1.27 | 1.15 | 1.38 |
| Additive | 1.29 | 1.17 | 1.41 |
| Disjunctive | 1.25 | 1.14 | 1.38 |

*Note: Contrast: (Self-relevant) / (Self-irrelevant)*

**Table I.** Posterior estimates for relative allocation in all experimental conditions in Exp 2.

| Task | Self-relevance | response | lower.HPD | upper.HPD |
| --- | --- | --- | --- | --- |
| Simple | Self-relevant | 0.56 | 0.54 | 0.58 |
| Additive | Self-relevant | 0.56 | 0.54 | 0.58 |
| Disjunctive | Self-relevant | 0.56 | 0.53 | 0.58 |
| Simple | Self-irrelevant | 0.50 | 0.48 | 0.52 |
| Additive | Self-irrelevant | 0.50 | 0.48 | 0.52 |
| Disjunctive | Self-irrelevant | 0.50 | 0.48 | 0.52 |
