# Supplementary material for: Self-allocation bias in performance-based cooperative decisions is driven by self-interest rather than distorted performance encoding
Source: PLoS Biol. 2026 Mar 26;24(3):e3003694. doi: 10.1371/journal.pbio.3003694 (PMC13020808; doi:10.1371/journal.pbio.3003694)
Supplement: S2 Appendix — (DOCX) [file pbio.3003694.s009.docx]

# **S2 Appendix**

All statistical analysis related to the model (*Formular 2*) testing the effect of self-relevance and collective task criteria on the relationship between SVO and relative allocation in the combined sample, or Experiment 1 and 2 respectively.

*Combined experiments*

**Table A.** The effect of self-relevance and collective task criteria on the relationship between SVO and relative allocation.

|  | Estimate | Est.Error | l-95% CI | u-95% CI | Rhat | Bulk_ESS | Tail_ESS |
| --- | --- | --- | --- | --- | --- | --- | --- |
| Intercept | 0.2 | 0.02 | 0.15 | 0.24 | 1 | 5139.96 | 8248.27 |
| taskAdditive | 0.03 | 0.03 | -0.03 | 0.08 | 1 | 8813.96 | 10883.75 |
| taskDisjunctive | 0.02 | 0.03 | -0.04 | 0.07 | 1 | 8857.45 | 10442 |
| conditionSelf-irrelevant | -0.2 | 0.03 | -0.26 | -0.15 | 1 | 7235.54 | 10354.04 |
| SVOscore_z | -0.17 | 0.03 | -0.22 | -0.12 | 1 | 4293.26 | 7312.62 |
| taskAdditive:conditionSelf-irrelevant | -0.03 | 0.04 | -0.1 | 0.05 | 1 | 7576.3 | 9929.95 |
| taskDisjunctive:conditionSelf-irrelevant | -0.02 | 0.04 | -0.09 | 0.06 | 1 | 7755.34 | 10185.42 |
| taskAdditive:SVOscore_z | 0.01 | 0.03 | -0.04 | 0.07 | 1 | 7439.54 | 9924.89 |
| taskDisjunctive:SVOscore_z | 0.02 | 0.03 | -0.04 | 0.07 | 1 | 7001.73 | 10201.67 |
| conditionSelf-irrelevant:SVOscore_z | 0.16 | 0.03 | 0.1 | 0.21 | 1 | 5771.32 | 9635.6 |
| taskAdditive:conditionSelf-irrelevant:SVOscore_z | 0 | 0.04 | -0.08 | 0.08 | 1 | 7208.32 | 10447.05 |
| taskDisjunctive:conditionSelf-irrelevant:SVOscore_z | 0 | 0.04 | -0.07 | 0.08 | 1 | 6482.8 | 9644.81 |

**Table B.** Post-hoc pairwise tests for the SVO slope in self-relevant vs. self-irrelevant conditions.

| Task | odds.ratio | lower.HPD | upper.HPD |
| --- | --- | --- | --- |
| Simple | -0.16 | -0.21 | -0.10 |
| Additive | -0.16 | -0.22 | -0.11 |
| Disjunctive | -0.16 | -0.22 | -0.11 |

*Note: Contrast: (Self-relevant) / (Self-irrelevant)*

**Table C.** Posterior estimates for the SVO slope on relative allocation in all experimental conditions.

| Self-relevance | Task | SVOscore_z.trend | lower.HPD | upper.HPD |
| --- | --- | --- | --- | --- |
| Self-relevant | Simple | -0.17 | -0.22 | -0.12 |
| Self-irrelevant | Simple | -0.01 | -0.06 | 0.04 |
| Self-relevant | Additive | -0.16 | -0.2 | -0.11 |
| Self-irrelevant | Additive | 0 | -0.04 | 0.05 |
| Self-relevant | Disjunctive | -0.15 | -0.2 | -0.1 |
| Self-irrelevant | Disjunctive | 0.01 | -0.04 | 0.06 |

*Experiment 1*

**Table D.** The effect of self-relevance and collective task criteria on the relationship between SVO and relative allocation in Exp 1.

|  | Estimate | Est.Error | l-95% CI | u-95% CI | Rhat | Bulk_ESS | Tail_ESS |
| --- | --- | --- | --- | --- | --- | --- | --- |
| Intercept | 0.2 | 0.03 | 0.14 | 0.27 | 1 | 5166.56 | 9025.43 |
| taskAdditive | 0.04 | 0.03 | -0.03 | 0.1 | 1 | 7780.46 | 10290.58 |
| taskDisjunctive | 0.03 | 0.03 | -0.04 | 0.1 | 1 | 7904.68 | 10886.69 |
| conditionSelf-irrelevant | -0.2 | 0.03 | -0.27 | -0.14 | 1 | 6816.12 | 10172.94 |
| SVOscore_z | -0.17 | 0.03 | -0.24 | -0.11 | 1 | 4133.02 | 7365.49 |
| taskAdditive:conditionSelf-irrelevant | -0.04 | 0.05 | -0.13 | 0.06 | 1 | 7253.65 | 10037.4 |
| taskDisjunctive:conditionSelf-irrelevant | -0.04 | 0.05 | -0.13 | 0.06 | 1 | 6946.84 | 9999.25 |
| taskAdditive:SVOscore_z | 0.01 | 0.04 | -0.06 | 0.08 | 1 | 6335.76 | 8604.59 |
| taskDisjunctive:SVOscore_z | 0.01 | 0.04 | -0.06 | 0.08 | 1 | 5978.44 | 8644.08 |
| conditionSelf-irrelevant:SVOscore_z | 0.16 | 0.04 | 0.1 | 0.23 | 1 | 5570.64 | 8183.36 |
| taskAdditive:conditionSelf-irrelevant:SVOscore_z | -0.02 | 0.05 | -0.11 | 0.08 | 1 | 6164.77 | 8803.95 |
| taskDisjunctive:conditionSelf-irrelevant:SVOscore_z | 0.01 | 0.05 | -0.08 | 0.11 | 1 | 5807.05 | 8954.88 |

**Table E.** Post-hoc pairwise tests for the SVO slope in self-relevant vs. self-irrelevant conditions in Exp 1.

| Task | odds.ratio | lower.HPD | upper.HPD |
| --- | --- | --- | --- |
| Simple | -0.16 | -0.23 | -0.09 |
| Additive | -0.15 | -0.22 | -0.08 |
| Disjunctive | -0.18 | -0.24 | -0.11 |

*Note: Contrast: (Self-relevant) / (Self-irrelevant)*

**Table F.** Posterior estimates for the SVO slope on relative allocation in all experimental conditions in Exp 1.

| Self-relevance | Task | SVOscore_z.trend | lower.HPD | upper.HPD |
| --- | --- | --- | --- | --- |
| Self-relevant | Simple | -0.17 | -0.24 | -0.11 |
| Self-irrelevant | Simple | -0.01 | -0.07 | 0.05 |
| Self-relevant | Additive | -0.16 | -0.22 | -0.1 |
| Self-irrelevant | Additive | -0.01 | -0.07 | 0.05 |
| Self-relevant | Disjunctive | -0.16 | -0.23 | -0.1 |
| Self-irrelevant | Disjunctive | 0.02 | -0.05 | 0.08 |

*Experiment 2*

**Table G.** The effect of self-relevance and collective task criteria on the relationship between SVO and relative allocation in Exp 2.

|  | Estimate | Est.Error | l-95% CI | u-95% CI | Rhat | Bulk_ESS | Tail_ESS |
| --- | --- | --- | --- | --- | --- | --- | --- |
| Intercept | 0.19 | 0.04 | 0.1 | 0.28 | 1 | 5644.6 | 9342.01 |
| taskAdditive | 0 | 0.05 | -0.09 | 0.1 | 1 | 8931.32 | 11807.34 |
| taskDisjunctive | 0 | 0.05 | -0.1 | 0.1 | 1 | 9073.69 | 11488.2 |
| conditionSelf-irrelevant | -0.2 | 0.05 | -0.3 | -0.1 | 1 | 7761.17 | 10544.91 |
| SVOscore_z | -0.16 | 0.05 | -0.25 | -0.07 | 1 | 5240.42 | 9212.93 |
| taskAdditive:conditionSelf-irrelevant | 0 | 0.07 | -0.13 | 0.14 | 1 | 8125.85 | 10639.36 |
| taskDisjunctive:conditionSelf-irrelevant | 0.01 | 0.07 | -0.13 | 0.14 | 1 | 8052.83 | 10858.45 |
| taskAdditive:SVOscore_z | 0 | 0.05 | -0.1 | 0.1 | 1 | 7539.69 | 10754.78 |
| taskDisjunctive:SVOscore_z | 0.02 | 0.05 | -0.08 | 0.12 | 1 | 7505.84 | 9988.53 |
| conditionSelf-irrelevant:SVOscore_z | 0.15 | 0.05 | 0.05 | 0.25 | 1 | 6288.02 | 9512.06 |
| taskAdditive:conditionSelf-irrelevant:SVOscore_z | 0.04 | 0.07 | -0.09 | 0.19 | 1 | 7289.31 | 10349.5 |
| taskDisjunctive:conditionSelf-irrelevant:SVOscore_z | 0 | 0.07 | -0.14 | 0.14 | 1 | 6747.82 | 9818.65 |

**Table H**. Post-hoc pairwise tests for the SVO slope in self-relevant vs. self-irrelevant conditions in Exp 2.

| Task | odds.ratio | lower.HPD | upper.HPD |
| --- | --- | --- | --- |
| Simple | -0.15 | -0.25 | -0.05 |
| Additive | -0.19 | -0.29 | -0.10 |
| Disjunctive | -0.15 | -0.25 | -0.05 |

*Note: Contrast: (Self-relevant) / (Self-irrelevant)*

**Table I.** Posterior estimates for the SVO slope on relative allocation in all experimental conditions in Exp 2.

| Self-relevance | Task | SVOscore_z.trend | lower.HPD | upper.HPD |
| --- | --- | --- | --- | --- |
| Self-relevant | Simple | -0.16 | -0.25 | -0.07 |
| Self-irrelevant | Simple | -0.01 | -0.1 | 0.07 |
| Self-relevant | Additive | -0.16 | -0.25 | -0.07 |
| Self-irrelevant | Additive | 0.03 | -0.06 | 0.11 |
| Self-relevant | Disjunctive | -0.14 | -0.23 | -0.05 |
| Self-irrelevant | Disjunctive | 0.01 | -0.08 | 0.09 |
