# Supplementary material for: Self-allocation bias in performance-based cooperative decisions is driven by self-interest rather than distorted performance encoding
Source: PLoS Biol. 2026 Mar 26;24(3):e3003694. doi: 10.1371/journal.pbio.3003694 (PMC13020808; doi:10.1371/journal.pbio.3003694)
Supplement: S3 Appendix — (DOCX) [file pbio.3003694.s010.docx]

# **S3 Appendix**

All statistical analysis related to the model (*Formular 2*) testing the effect of self-relevance and collective task criteria on the relationship between relative performance and relative allocation in the combined sample, or Experiment 1 and 2 respectively.

*Combined experiments*

**Table A.** The effect of self-relevance and collective task criteria on the relationship between relative performance and relative allocation.

|  | Estimate | Est.Error | l-95% CI | u-95% CI | Rhat | Bulk_ESS | Tail_ESS |
| --- | --- | --- | --- | --- | --- | --- | --- |
| Intercept | -1.07 | 0.04 | -1.14 | -1 | 1 | 2950.34 | 5980.28 |
| performance_ratio | 2.64 | 0.05 | 2.55 | 2.73 | 1 | 9973.81 | 11875.91 |
| taskAdditive | -0.11 | 0.03 | -0.17 | -0.04 | 1 | 10396.95 | 11666.98 |
| taskDisjunctive | -0.42 | 0.04 | -0.5 | -0.34 | 1 | 9842.96 | 10569.48 |
| conditionSelf-irrelevant | -0.54 | 0.03 | -0.59 | -0.48 | 1 | 11013.72 | 11830.08 |
| performance_ratio:taskAdditive | 0.22 | 0.06 | 0.1 | 0.34 | 1 | 10241.04 | 11405.07 |
| performance_ratio:taskDisjunctive | 0.85 | 0.07 | 0.71 | 1 | 1 | 9995.38 | 11142.02 |
| performance_ratio:conditionSelf-irrelevant | 0.57 | 0.05 | 0.46 | 0.67 | 1 | 10910.15 | 10977.98 |

**Table B.** Posterior estimates for the slope of relative performance on relative allocation in self-relevance conditions.

| Self-relevance | performance_ratio.trend | lower.HPD | upper.HPD |
| --- | --- | --- | --- |
| Self-relevant | 3 | 2.92 | 3.07 |
| Self-irrelevant | 3.56 | 3.49 | 3.65 |

**Table C.** Post-hoc pairwise tests for slopes of relative performance in three task criteria.

| Contrasts | estimate | lower.HPD | upper.HPD |
| --- | --- | --- | --- |
| Simple - Additive | -0.22 | -0.34 | -0.10 |
| Simple - Disjunctive | -0.85 | -1.00 | -0.71 |
| Additive - Disjunctive | -0.63 | -0.78 | -0.48 |

*Experiment 1*

**Table D.** The effect of self-relevance and collective task criteria on the relationship between relative performance and relative allocation in Exp 1.

|  | Estimate | Est.Error | l-95% CI | u-95% CI | Rhat | Bulk_ESS | Tail_ESS |
| --- | --- | --- | --- | --- | --- | --- | --- |
| Intercept | -1.11 | 0.04 | -1.2 | -1.02 | 1 | 2536.13 | 5454.42 |
| performance_ratio | 2.67 | 0.06 | 2.56 | 2.79 | 1 | 6296.28 | 9282.11 |
| taskAdditive | -0.1 | 0.04 | -0.17 | -0.02 | 1 | 6382.87 | 8399.2 |
| taskDisjunctive | -0.36 | 0.05 | -0.45 | -0.27 | 1 | 6495.93 | 8753.69 |
| conditionSelf-irrelevant | -0.46 | 0.03 | -0.53 | -0.4 | 1 | 7651.36 | 9238.68 |
| performance_ratio:taskAdditive | 0.22 | 0.07 | 0.08 | 0.36 | 1 | 6248.01 | 8702.45 |
| performance_ratio:taskDisjunctive | 0.75 | 0.09 | 0.58 | 0.91 | 1 | 6374.58 | 8632.18 |
| performance_ratio:conditionSelf-irrelevant | 0.46 | 0.06 | 0.34 | 0.59 | 1 | 7648.46 | 9447.22 |

**Table E.** Posterior estimates for the slope of relative performance on relative allocation in self-relevance conditions in Exp 1.

| Self-relevance | performance_ratio.trend | lower.HPD | upper.HPD |
| --- | --- | --- | --- |
| Self-relevant | 2.99 | 2.9 | 3.08 |
| Self-irrelevant | 3.46 | 3.36 | 3.55 |

**Table F.** Post-hoc pairwise tests for slopes of relative performance in three task criteria in Exp 1.

| Contrasts | estimate | lower.HPD | upper.HPD |
| --- | --- | --- | --- |
| Simple - Additive | -0.22 | -0.36 | -0.08 |
| Simple - Disjunctive | -0.75 | -0.92 | -0.58 |
| Additive - Disjunctive | -0.53 | -0.70 | -0.36 |

*Experiment 2*

**Table G.** The effect of self-relevance and collective task criteria on the relationship between relative performance and relative allocation in Exp 2.

|  | Estimate | Est.Error | l-95% CI | u-95% CI | Rhat | Bulk_ESS | Tail_ESS |
| --- | --- | --- | --- | --- | --- | --- | --- |
| Intercept | -0.99 | 0.06 | -1.1 | -0.87 | 1 | 2759.34 | 5358.03 |
| performance_ratio | 2.58 | 0.08 | 2.41 | 2.74 | 1 | 6933.11 | 10304.46 |
| taskAdditive | -0.13 | 0.06 | -0.24 | -0.02 | 1 | 7964.62 | 9809.29 |
| taskDisjunctive | -0.61 | 0.08 | -0.77 | -0.46 | 1 | 8137.29 | 9848.72 |
| conditionSelf-irrelevant | -0.68 | 0.05 | -0.78 | -0.58 | 1 | 8712.54 | 10695.91 |
| performance_ratio:taskAdditive | 0.23 | 0.1 | 0.02 | 0.43 | 1 | 7683.04 | 9668.95 |
| performance_ratio:taskDisjunctive | 1.21 | 0.15 | 0.93 | 1.51 | 1 | 8087.86 | 9913.4 |
| performance_ratio:conditionSelf-irrelevant | 0.77 | 0.1 | 0.58 | 0.96 | 1 | 8588.71 | 9522.67 |

**Table H.** Posterior estimates for the slope of relative performance on relative allocation in self-relevance conditions in Exp 2.

| Self-relevance | performance_ratio.trend | lower.HPD | upper.HPD |
| --- | --- | --- | --- |
| Self-relevant | 3.06 | 2.91 | 3.2 |
| Self-irrelevant | 3.83 | 3.68 | 3.98 |

**Table I.** Post-hoc pairwise tests for slopes of relative performance in three task criteria in Exp 2.

| Contrasts | estimate | lower.HPD | upper.HPD |
| --- | --- | --- | --- |
| Simple - Additive | -0.23 | -0.43 | -0.02 |
| Simple - Disjunctive | -1.21 | -1.50 | -0.92 |
| Additive - Disjunctive | -0.98 | -1.30 | -0.69 |
