# Supplementary material for: Self-allocation bias in performance-based cooperative decisions is driven by self-interest rather than distorted performance encoding
Source: PLoS Biol. 2026 Mar 26;24(3):e3003694. doi: 10.1371/journal.pbio.3003694 (PMC13020808; doi:10.1371/journal.pbio.3003694)
Supplement: S4 Appendix — (DOCX) [file pbio.3003694.s011.docx]

# **S4 Appendix**

All statistical analysis related to the model (*Formular 4*) testing the effect of contribution on self-allocation bias.in the combined sample, or Experiment 1 and 2 respectively.

**Table A.** Trial number distribution according to contribution in self- relevant/irrelevant conditions.

|  | Player1 only | | | Both | | | Player2 only | | |
| --- | --- | --- | --- | --- | --- | --- | --- | --- | --- |
|  | Simple | Additive | Disjunctive | Simple | Additive | Disjunctive | Simple | Additive | Disjunctive |
| Exp 1 | 0 | 8 | 10 | 32 | 16 | 12 | 0 | 8 | 10 |
| Exp 2 | 0 | 3 | 5 | 18 | 12 | 8 | 0 | 3 | 5 |

*Combined experiments*

**Table B.** The effect of contribution on self-allocation bias.

|  | Estimate | | Est.Error | | l-95% CI | | u-95% CI | | Rhat | | Bulk_ESS | | Tail_ESS | |
| --- | --- | --- | --- | --- | --- | --- | --- | --- | --- | --- | --- | --- | --- | --- |
| Intercept | 0.23 | | 0.04 | | 0.15 | | 0.32 | | 1 | | 8428.25 | | 10274.21 | |
| taskAdditive | 0.01 | | 0.05 | | -0.08 | | 0.1 | | 1 | | 14474.14 | | 12797.36 | |
| taskDisjunctive | -0.01 | | 0.05 | | -0.1 | | 0.09 | | 1 | | 14688.53 | | 13739.61 | |
| conditionSelf-irrelevant | -0.24 | | 0.05 | | -0.33 | | -0.14 | | 1 | | 12754.83 | | 12991.39 | |
|  | | Estimate | | Est.Error | | l-95% CI | | u-95% CI | | Rhat | | Bulk_ESS | | Tail_ESS |
| Intercept | | 0.05 | | 0.01 | | 0.02 | | 0.08 | | 1.01 | | 871.32 | | 1613.02 |
| contributionBoth | | 0.02 | | 0.01 | | 0.01 | | 0.03 | | 1 | | 17215.15 | | 12270.88 |
| contributionPlayer2only | | 0.06 | | 0.01 | | 0.05 | | 0.08 | | 1 | | 16997.35 | | 11874.98 |

**Table C.** Posterior estimates for the effect of contribution on self-allocation bias.

| Contribution | emmean | lower.HPD | upper.HPD |
| --- | --- | --- | --- |
| Player1 only | 0.05 | 0.02 | 0.08 |
| Both | 0.07 | 0.05 | 0.1 |
| Player2 only | 0.11 | 0.09 | 0.14 |

**Table D.** Post-hoc pairwise tests for self-allocation bias in three contribution structures.

| Contrasts | estimate | lower.HPD | upper.HPD |
| --- | --- | --- | --- |
| Player1 only - Both | -0.02 | -0.03 | -0.01 |
| Player1 only - Player2 only | -0.06 | -0.08 | -0.05 |
| Both - Player2 only | -0.04 | -0.05 | -0.03 |

**Table E.** Posterior estimates for the slope of SVO on self-allocation bias in three contribution structures.

| Contribution | SVOscore_z.trend | lower.HPD | upper.HPD |
| --- | --- | --- | --- |
| Player1 only | -0.03 | -0.05 | 0 |
| Both | -0.06 | -0.09 | -0.04 |
| Player2 only | -0.09 | -0.11 | -0.07 |

**Table F.** Post-hoc pairwise tests for the slope of SVO on self-allocation bias in three contribution structures.

| Contrasts | estimate | lower.HPD | upper.HPD |
| --- | --- | --- | --- |
| Player1 only - Both | 0.03 | 0.02 | 0.04 |
| Player1 only - Player2 only | 0.06 | 0.05 | 0.07 |
| Both - Player2 only | 0.03 | 0.02 | 0.04 |

*Experiment 1*

**Table G.** The effect of contribution on self-allocation bias in Exp 1.

|  | Estimate | Est.Error | l-95% CI | u-95% CI | Rhat | Bulk_ESS | Tail_ESS |
| --- | --- | --- | --- | --- | --- | --- | --- |
| Intercept | 0.05 | 0.02 | 0.01 | 0.08 | 1.01 | 866.19 | 1629.79 |
| contributionBoth | 0.01 | 0.01 | 0 | 0.03 | 1 | 9717.56 | 10713.2 |
| contributionPlayer2only | 0.05 | 0.01 | 0.03 | 0.07 | 1 | 8828.52 | 10507.8 |

**Table H.** Posterior estimates for the effect of contribution on self-allocation bias in Exp 1.

| Contribution | emmean | lower.HPD | upper.HPD |
| --- | --- | --- | --- |
| Player1 only | 0.05 | 0.01 | 0.08 |
| Both | 0.06 | 0.02 | 0.09 |
| Player2 only | 0.1 | 0.06 | 0.13 |

**Table I.** Post-hoc pairwise tests for self-allocation bias in three contribution structures in Exp 1.

| Contrasts | estimate | lower.HPD | upper.HPD |
| --- | --- | --- | --- |
| Player1 only - Both | -0.01 | -0.03 | -0.0005 |
| Player1 only - Player2 only | -0.05 | -0.07 | -0.03 |
| Both - Player2 only | -0.04 | -0.05 | -0.02 |

**Table J.** Posterior estimates for the slope of SVO on self-allocation bias in three contribution structures in Exp 1.

| Contribution | SVOscore_z.trend | lower.HPD | upper.HPD |
| --- | --- | --- | --- |
| Player1 only | -0.02 | -0.05 | 0.01 |
| Both | -0.06 | -0.09 | -0.03 |
| Player2 only | -0.08 | -0.11 | -0.05 |

**Table K.** Post-hoc pairwise tests for the slope of SVO on self-allocation bias in three contribution structures in Exp 1.

| Contrasts | estimate | lower.HPD | upper.HPD |
| --- | --- | --- | --- |
| Player1 only - Both | 0.04 | 0.02 | 0.05 |
| Player1 only - Player2 only | 0.06 | 0.04 | 0.08 |
| Both - Player2 only | 0.02 | 0.01 | 0.04 |

*Experiment 2*

**Table L.** The effect of contribution on self-allocation bias in Exp 2.

|  | Estimate | Est.Error | l-95% CI | u-95% CI | Rhat | Bulk_ESS | Tail_ESS |
| --- | --- | --- | --- | --- | --- | --- | --- |
| Intercept | 0.05 | 0.02 | 0 | 0.09 | 1.01 | 908.81 | 1841.55 |
| contributionBoth | 0.04 | 0.01 | 0.02 | 0.06 | 1 | 7764.71 | 9871.68 |
| contributionPlayer2only | 0.1 | 0.01 | 0.07 | 0.12 | 1 | 7609.06 | 8750.91 |

**Table M.** Posterior estimates for the effect of contribution on self-allocation bias in Exp 2.

| Contribution | emmean | lower.HPD | upper.HPD |
| --- | --- | --- | --- |
| Player1 only | 0.05 | 0 | 0.09 |
| Both | 0.09 | 0.04 | 0.13 |
| Player2 only | 0.14 | 0.1 | 0.19 |

**Table N.** Post-hoc pairwise tests for self-allocation bias in three contribution structures in Exp 2.

| Contrasts | estimate | lower.HPD | upper.HPD |
| --- | --- | --- | --- |
| Player1 only - Both | -0.04 | -0.06 | -0.02 |
| Player1 only - Player2 only | -0.10 | -0.12 | -0.07 |
| Both - Player2 only | -0.06 | -0.08 | -0.04 |

**Table O.** Posterior estimates for the slope of SVO on self-allocation bias in three contribution structures in Exp 2.

| Contribution | SVOscore_z.trend | lower.HPD | upper.HPD |
| --- | --- | --- | --- |
| Player1 only | -0.02 | -0.05 | -0.09 |
| Both | -0.06 | -0.07 | -0.11 |
| Player2 only | -0.08 | -0.1 | -0.14 |

**Table P.** Post-hoc pairwise tests for the slope of SVO on self-allocation bias in three contribution structures in Exp 2.

| Contrasts | estimate | lower.HPD | upper.HPD |
| --- | --- | --- | --- |
| Player1 only - Both | 0.02 | 0.003 | 0.04 |
| Player1 only - Player2 only | 0.05 | 0.02 | 0.08 |
| Both - Player2 only | 0.03 | 0.01 | 0.05 |
