# Supplementary material for: Self-allocation bias in performance-based cooperative decisions is driven by self-interest rather than distorted performance encoding
Source: PLoS Biol. 2026 Mar 26;24(3):e3003694. doi: 10.1371/journal.pbio.3003694 (PMC13020808; doi:10.1371/journal.pbio.3003694)
Supplement: S5 Appendix — (DOCX) [file pbio.3003694.s012.docx]

# **S5 Appendix**

GLM results

**Table A.** GLM1: Factorial design of self-relevance and contribution with relative performance asparametric modulator.

|  | Side | x | y | z | Cluster size | p(FWEc) | Peak *T/F* |
| --- | --- | --- | --- | --- | --- | --- | --- |
| **Onset: Self-relevant > Self-irrelevant** | |  |  |  |  |  | *T* |
| R Superior Frontal Gyrus/mPFC | L/R | 18 | 54 | 24 | 2788 | <0.0001 | 8.01 |
| R Insula Lobe/Anterior Insula | R | 34 | 16 | -10 | 212 | 0.002 | 5.93 |
| R Angular Gyrus/TPJ | R | 46 | -56 | 32 | 151 | 0.014 | 4.25 |
| **Parametric modulation: Contribution** | | | | | |  | *F* |
| R Superior Medial Gyrus/dmPFC | R | 12 | 28 | 52 | 524 | <0.0001 | 28.05 |
| L Middle Orbital Gyrus/lOFC | L | -42 | 50 | -6 | 116 | 0.024 | 23.56 |
| R Middle Temporal Gyrus (MTG) | R | 64 | -38 | -4 | 108 | 0.033 | 21.33 |
| L Middle Orbital Gyrus/lOFC | R | 40 | 48 | -12 | 293 | <0.0001 | 19.12 |
| R Angular Gyrus/TPJ | R | 44 | -64 | 42 | 591 | <0.0001 | 18.86 |
| L Angular Gyrus/TPJ | L | -56 | -64 | 30 | 124 | 0.018 | 12.99 |

*Note: No clusters survived in other contrasts after FWEc correction.*

**Table B.** GLM2: Factorial design of self-relevance with relative allocation as parametric modulator.

|  | Side | x | y | z | Cluster size | p(FWEc) | Peak *T/F* |
| --- | --- | --- | --- | --- | --- | --- | --- |
| **Onset: Self-relevant > Self-irrelevant** | |  |  |  |  |  | *T* |
| R Superior Frontal Gyrus/mPFC | L/R | 18 | 28 | 54 | 3535 | <0.0001 | 6.92 |
| R Angular Gyrus/TPJ | R | 50 | -60 | 32 | 707 | <0.0001 | 5.98 |
| L Insula Lobe/Anterior Insula | L | -30 | 16 | 4 | 212 | 0.003 | 5.91 |
| R Insula Lobe/Anterior Insula | R | 34 | 16 | -14 | 366 | <0.0001 | 5.79 |
| L Angular Gyrus/TPJ | L | -46 | -62 | 34 | 128 | 0.035 | 4.72 |

*Note: No clusters survived in other contrasts after FWEc correction.*
