# Supplementary material for: Self-allocation bias in performance-based cooperative decisions is driven by self-interest rather than distorted performance encoding
Source: PLoS Biol. 2026 Mar 26;24(3):e3003694. doi: 10.1371/journal.pbio.3003694 (PMC13020808; doi:10.1371/journal.pbio.3003694)
Supplement: S6 Appendix — (DOCX) [file pbio.3003694.s013.docx]

# **S6 Appendix**

**Table A:** Statistical tests for the difference in beta estimates from ROI at three contribution structures.

| ROI | Contribution | n1 | n2 | t | df | p | conf.low | conf.high | p.adj | signif |
| --- | --- | --- | --- | --- | --- | --- | --- | --- | --- | --- |
| dmPFC | Player1 only vs. Both | 34 | 34 | -4.20 | 33 | <0.001 | -2.14 | -0.74 | <0.001 | *** |
| dmPFC | Player1 only vs. Player2 only | 34 | 34 | -4.50 | 33 | <0.001 | -3.36 | -1.27 | <0.001 | *** |
| dmPFC | Both vs. Player2 only | 34 | 34 | -2.28 | 33 | 0.029 | -1.64 | -0.09 | 0.088 | ns |
| Left lOFC | Player1 only vs. Both | 34 | 34 | -3.70 | 33 | <0.001 | -2.26 | -0.65 | 0.002 | ** |
| Left lOFC | Player1 only vs. Player2 only | 34 | 34 | -4.21 | 33 | <0.001 | -3.66 | -1.27 | <0.001 | *** |
| Left lOFC | Both vs. Player2 only | 34 | 34 | -1.80 | 33 | 0.081 | -2.16 | 0.133 | 0.24 | ns |
| Right lOFC | Player1 only vs. Both | 34 | 34 | -4.50 | 33 | <0.001 | -2.09 | -0.79 | <0.001 | *** |
| Right lOFC | Player1 only vs. Player2 only | 34 | 34 | -4.74 | 33 | <0.001 | -3.22 | -1.29 | <0.001 | *** |
| Right lOFC | Both vs. Player2 only | 34 | 34 | -2.18 | 33 | 0.037 | -1.57 | -0.05 | 0.11 | ns |
| Right MTG | Player1 only vs. Both | 34 | 34 | -4.12 | 33 | <0.001 | -2.61 | -0.89 | <0.001 | *** |
| Right MTG | Player1 only vs. Player2 only | 34 | 34 | -4.40 | 33 | <0.001 | -3.29 | -1.21 | <0.001 | *** |
| Right MTG | Both vs. Player2 only | 34 | 34 | -1.10 | 33 | 0.28 | -1.43 | 0.42 | 0.83 | ns |
| Left TPJ | Player1 only vs. Both | 34 | 34 | -3.42 | 33 | 0.002 | -3.35 | -0.85 | 0.005 | ** |
| Left TPJ | Player1 only vs. Player2 only | 34 | 34 | -3.71 | 33 | <0.001 | -3.85 | -1.12 | 0.002 | ** |
| Left TPJ | Both vs. Player2 only | 34 | 34 | -0.57 | 33 | 0.58 | -1.77 | 1.00 | 1 | ns |
| Right TPJ | Player1 only vs. Both | 34 | 34 | -4.58 | 33 | <0.001 | -2.80622 | -1.08 | <0.001 | *** |
| Right TPJ | Player1 only vs. Player2 only | 34 | 34 | -3.75 | 33 | <0.001 | -3.86 | -1.14 | 0.002 | ** |
| Right TPJ | Both vs. Player2 only | 34 | 34 | -1.15 | 33 | 0.26 | -1.54 | 0.42 | 0.77 | ns |
