# Supplementary material for: Self-allocation bias in performance-based cooperative decisions is driven by self-interest rather than distorted performance encoding
Source: PLoS Biol. 2026 Mar 26;24(3):e3003694. doi: 10.1371/journal.pbio.3003694 (PMC13020808; doi:10.1371/journal.pbio.3003694)
Supplement: S7 Appendix — (DOCX) [file pbio.3003694.s014.docx]

# **S7 Appendix**

All statistical tests for the additional experiment 3

**Table A.** The effect of self-relevance in performance source, allocation target, and collective task criteria on relative allocation in Exp 3.

|  | Estimate | Est.Error | l-95% CI | u-95% CI | Rhat | Bulk_ESS | Tail_ESS |
| --- | --- | --- | --- | --- | --- | --- | --- |
| Intercept | 0.2 | 0.03 | 0.14 | 0.26 | 1 | 2490.79 | 5032.77 |
| taskAdditive | 0.01 | 0.03 | -0.05 | 0.07 | 1 | 6669.4 | 9519.95 |
| taskDisjunctive | -0.01 | 0.03 | -0.07 | 0.05 | 1 | 6366.8 | 9279.85 |
| p_sourceSelf-irrelevant | 0.01 | 0.03 | -0.05 | 0.07 | 1 | 5467.17 | 8922.5 |
| a_targetSelf-irrelevant | -0.14 | 0.03 | -0.19 | -0.08 | 1 | 5606.7 | 8905.31 |
| taskAdditive:p_sourceSelf-irrelevant | -0.01 | 0.04 | -0.09 | 0.08 | 1 | 6083.86 | 9607.8 |
| taskDisjunctive:p_sourceSelf-irrelevant | 0 | 0.04 | -0.08 | 0.09 | 1 | 5528.16 | 9215.45 |
| taskAdditive:a_targetSelf-irrelevant | -0.02 | 0.04 | -0.11 | 0.06 | 1 | 5961.17 | 8817.21 |
| taskDisjunctive:a_targetSelf-irrelevant | 0 | 0.04 | -0.09 | 0.08 | 1 | 5909.21 | 8945.91 |
| p_sourceSelf-irrelevant:a_targetSelf-irrelevant | -0.03 | 0.04 | -0.12 | 0.05 | 1 | 4912.84 | 8556.83 |
| taskAdditive:p_sourceSelf-irrelevant:a_targetSelf-irrelevant | 0 | 0.06 | -0.11 | 0.12 | 1 | 5548.9 | 8971.97 |
| taskDisjunctive:p_sourceSelf-irrelevant:a_targetSelf-irrelevant | 0 | 0.06 | -0.12 | 0.12 | 1 | 5382.94 | 8157.89 |

**Table B.** Post-hoc pairwise tests for relative allocation in self-relevant vs. self-irrelevant conditions in Exp 3.

| Contrast | estimate | lower.HPD | upper.HPD |
| --- | --- | --- | --- |
| a_target = Self-relevant (SforS-OforS) | 0.99 | 0.96 | 1.03 |
| a_target = Self-irrelevant (SforO-OforO) | 1.02 | 0.99 | 1.06 |
| p_source = Self-relevant (SforS-SforO) | 1.15 | 1.11 | 1.10 |
| p_source = Self-irrelevant (OforS-OforO) | 1.19 | 1.15 | 1.24 |

**Table C.** Posterior estimates for relative allocation in all experimental conditions in Exp 3..

| Condition | response | lower.HPD | upper.HPD |
| --- | --- | --- | --- |
| SforS | 0.55 | 0.54 | 0.56 |
| SforO | 0.51 | 0.50 | 0.53 |
| OforS | 0.55 | 0.54 | 0.56 |
| OforO | 0.51 | 0.50 | 0.52 |

**Table D.** The effect of allocation target on the relationship between SVO and relative allocation in Exp 3.

|  | Estimate | Est.Error | l-95% CI | u-95% CI | Rhat | Bulk_ESS | Tail_ESS |
| --- | --- | --- | --- | --- | --- | --- | --- |
| Intercept | 0.22 | 0.02 | 0.17 | 0.26 | 1 | 1921.13 | 4195.34 |
| a_targetSelf-irrelevant | -0.17 | 0.01 | -0.2 | -0.15 | 1 | 20231.31 | 12640.05 |
| SVOscore_z | -0.11 | 0.02 | -0.16 | -0.07 | 1 | 2244.91 | 4765.54 |
| a_targetSelf-irrelevant:SVOscore_z | 0.08 | 0.01 | 0.06 | 0.11 | 1 | 16627.48 | 11591.54 |

**Table E.** The effect of allocation target on the relationship between relative performance and relative allocation in Exp 3.

|  | Estimate | Est.Error | l-95% CI | u-95% CI | Rhat | Bulk_ESS | Tail_ESS |
| --- | --- | --- | --- | --- | --- | --- | --- |
| Intercept | -1.11 | 0.04 | -1.19 | -1.04 | 1.01 | 717.04 | 1493.23 |
| performance_ratio | 2.74 | 0.03 | 2.68 | 2.8 | 1 | 8519.31 | 11095.15 |
| a_targetSelf-irrelevant | -0.34 | 0.02 | -0.38 | -0.29 | 1 | 7097.08 | 9765.29 |
| performance_ratio:a_targetSelf-irrelevant | 0.3 | 0.04 | 0.21 | 0.39 | 1 | 7025 | 9523.25 |

**Table F.** Post-hoc pairwise tests for self-allocation bias in three contribution structures in Exp 3.

| Contrasts | estimate | lower.HPD | upper.HPD |
| --- | --- | --- | --- |
| Player1 only - Both | -0.02 | -0.03 | -0.02 |
| Player1 only - Player2 only | -0.06 | -0.07 | -0.05 |
| Both - Player2 only | -0.04 | -0.04 | -0.03 |

**Table G.** Post-hoc pairwise tests for the slope of SVO on self-allocation bias in three contribution structures in Exp 3.

| Contrasts | estimate | lower.HPD | upper.HPD |
| --- | --- | --- | --- |
| Player1 only - Both | 0.01 | 0.001 | 0.02 |
| Player1 only - Player2 only | 0.02 | 0.01 | 0.03 |
| Both - Player2 only | 0.01 | 0.006 | 0.02 |
