# Supplementary material for: Self-allocation bias in performance-based cooperative decisions is driven by self-interest rather than distorted performance encoding
Source: PLoS Biol. 2026 Mar 26;24(3):e3003694. doi: 10.1371/journal.pbio.3003694 (PMC13020808; doi:10.1371/journal.pbio.3003694)
Supplement: Attachment — Submitted filename: PB20250822_REVIEWS_Rebuttal letter_final.docx [file pbio.3003694.s017.docx]

***Responses to reviewers***

Thank you for the reviewers’ thoughtful and constructive comments. Below is a brief summary of the major analyses and revisions made to substantially strengthen the manuscript:

First, we replaced the previous ANOVA analyses with Bayesian mixed-effects models, providing more robust results across experiments and addressing concerns about small sample sizes.

Second, we conducted two additional GLMs to clarify the fMRI findings and performed confirming ROI analyses.

Third, we performed a control analysis by excluding participants who expressed doubts under the believability manipulation. The main behavioral and fMRI findings remain consistent, while we have removed the exploratory covariate analysis that did not hold and caused confusion in the previous version.

In addition, we refined all figures to improve readability and revised the Introduction and Discussion sections to clarify the theoretical framework and strengthen the conceptual links to the broader literature.

Finally, we conducted additional research on the TPJ definition and labeling to ensure accurate anatomical characterization.

Detailed responses to each reviewer’s comment are provided below. All changes in the revised manuscript are highlighted in blue.

# **Reviewer #1**

In this study, Zhang and colleagues investigated how human participants distribute resources (money) between two individuals—either themselves and another person, or two other people—based on each individual's performance in a separate task. They found an allocation bias whereby participants tended to allocate more money to themselves than to others. The authors then examined the neural mechanisms underlying this biased allocation process.
The study is interesting and potentially informative. However, I have serious concerns regarding the study's design, the statistical analyses (both behavioral and fMRI), and certain aspects of the conclusions drawn. I believe the authors should be given an opportunity to resubmit, as my comments are all addressable. 
Below are my comments, which I hope the authors will find constructive and helpful.

***Overall response to Reviewer #1’s comment:*** We are grateful to Reviewer #1 for the supportive and constructive feedback, which has been instrumental in strengthening our manuscript. We have fully addressed all comments through substantial textual revisions, rigorous re-analyses of behavioral data using mixed-effects models, and additional neuroimaging analyses. Below, we provide a point-by-point response.

**Major comments:
#1-** The study is conceptually related to shared responsibility judgement, where people working in groups decide who to blame/credit for joint outcomes. This is very close to what this study investigates. However, never in the introduction or discussion is this link mentioned. In particular, some key references of the shared responsibility literature are missing. For example,
Xiang et al Cognition 2023 [https://www.sciencedirect.com/science/article/abs/pii/S001027723002433](https://www.sciencedirect.com/science/article/abs/pii/S0010027723002433#inbox/_blank)
Gerstenberg and Lagnado 2010. [https://psycnet.apa.org/record/2010-04148-013](https://psycnet.apa.org/record/2010-04148-013#inbox/_blank)
Mahmoodi et al. 2024. [https://www.sciencedirect.com/science/article/pii/S0896627324006494](https://www.sciencedirect.com/science/article/pii/S0896627324006494#inbox/_blank)
Gerstenberg et al. 2023.  [https://www.sciencedirect.com/science/article/abs/pii/S0010027723001336](https://www.sciencedirect.com/science/article/abs/pii/S0010027723001336#inbox/_blank)
 The introduction talks at length about experiments using games (game theory). I understand the link, the authors use one to study joint responsibility, but I do not see a clear conceptual link between this study and game theory.

***Response*:** We thank the reviewer for this excellent suggestion. We agree that linking our work to the literature on shared responsibility judgement provides a valuable conceptual framework. In the revised manuscript, we have now integrated this line of research into both the Introduction and Discussion sections, clarifying the conceptual link between our work and this field. As suggested, we have cited the key references (Xiang et al., 2023; Mahmoodi et al. 2024; Gerstenberg et al. 2023; etc.).

In the revised Introduction (Page 3, Line 56-60), we now position our study as bridging the gap between research on how people judge responsibility/contribution in groups and how they translate those judgements into concrete resource distributions. We introduce key references from the shared responsibility literature (Xiang et al., 2023; Gerstenberg & Lagnado, 2010; Mahmoodi et al., 2024; Gerstenberg et al., 2023) to establish this conceptual background.

In the revised Discussion (Page 13, Line 390-396), we return to this link, clarifying that the "self-allocation bias" we identify represents a self-serving distortion in the responsibility-to-reward transformation process.

Manuscript changes:

Revised Introduction (Page 3, Line 56-60): “The process of assessing each individual’s contributions is conceptually linked to judgments of shared responsibility, where individuals evaluate each member’s credit or blame for a group outcome based on their performance and the causal structure of the task (1–4). While research on shared responsibility has delineated the psychological processes of attribution (4–6), less is known about how these assessments are translated into resource distributions.” (Page 3, Line 91-93) “To bridge the psychological processes of contribution assessment with the economic problem of reward division, we turned to game theory.”

Revised Discussion (Page 13, Line 390-396): “While previous research has examined how people make self-other economic allocations in simplified paradigms (61,28,35) and how people evaluate individual and shared responsibility in group tasks (2–4), a critical gap remains in understanding how people integrate objective performance metrics and task contributions—factors central to real-world cooperation—into allocation decisions. The present study bridged this gap by investigating the cognitive and neural mechanisms underlying performance-based allocations in collective tasks.”

**#2-** The authors conducted linear regressions to examine the effect of relative performance on allocation across conditions (line 259 onward). They report using t-tests as their statistical test and then compared the resulting beta weights between conditions. This suggests that they did not use a mixed-effects linear regression. If this is the case, I strongly encourage the authors to reanalyze these data using a mixed-effects model, including condition as a fixed effect and testing the interaction between condition and relative performance. This approach would allow them to assess differences between conditions directly within a single model, while properly accounting for within-subject variability.
I do not expect this change to substantially alter the results, but it is important for ensuring that the findings generalize beyond the current sample. Simple t-tests only test for differences within the sample at hand, whereas mixed-effects modeling appropriately generalizes the inference to the broader population.

**#3-** Similarly, a major issue in the data analysis is the use of t-tests and ANOVAs instead of linear regression models. For example, in the behavioral data analysis (e.g., line 200 onward), the authors compared performance weights to zero and conducted ANOVAs to compare weights across conditions. However, the authors could—and in my view should—perform their analyses using a single linear model, with allocation as the dependent variable and relative performance and condition(s) as independent variables, including all relevant interaction terms. This would allow the authors to examine the effects of all key factors simultaneously and to test for interactions directly.
This approach is important because it can also be applied to the neuroimaging analysis. Instead of conducting GLMs based solely on factorial designs, the authors could use parametric designs incorporating allocation, condition, and their interaction as regressors. This would make it possible to identify the unique contribution of each factor and any differences between them. In contrast, the current analytical approach introduces unnecessary confusion and potential confounds. For instance, the authors report greater activity in the mPFC, TPJ, ACC, and insula in the self-relevant compared to the self-irrelevant condition. However, the behavioral results show that allocations were higher in the self-relevant condition. Therefore, this analysis does not clarify whether the reported neural differences reflect the parametric effect of allocation or simply self-relevance.
Relatedly, I am also concerned about the way the neural GLMs were implemented. In one GLM (line 337 onward), the authors included relative performance as a parametric modulator (PM) at the individual level, and allocation bias at the group-level GLM. This approach is confusing and raises questions about its rationale. If the goal is to examine the effect of relative performance (RP) on brain activity, the GLM should include RP as a PM without modulating the group level by allocation bias. Why should the neural representation of RP depend on allocation bias? This implies an assumption that the way people represent relative performance is conditional on their allocation bias, but the authors do not provide a clear justification for this. Why not first run a simpler GLM with only RP as the parametric modulator?
If the simpler GLM does not yield significant results, and the authors' approach does, this should be clearly reported and discussed. Such a result does not compromise the study—it strengthens it by demonstrating that the more complex model is justified.

***Response*:** We thank the reviewer for these critical and constructive methodological suggestions. In direct response, we have undertaken a comprehensive re-analysis of all behavioral data using mixed-effects models and two additional GLM analyses of the neuroimaging data, as recommended, and have updated the manuscript accordingly.

1) Comprehensive Re-analysis of Behavioral Data: We have completely replaced all previous t-test and ANOVA-based analyses with mixed-effects models.

Key Confirmation: We are pleased to confirm that all our key behavioral findings remain robust and statistically significant under this more rigorous analytical framework (revised Results: Page 6-10, Line 210-332; revised Methods: Page 18-19, Line 617-677).

Manuscript changes:

The "Behavioral results" and "Behavioral data analysis" sections have been thoroughly rewritten around these new models. All relevant figures and supplementary tables have also been updated. As the full set of results is extensive, we have not reprinted them there.

2) Neuroimaging Analyses:

Regarding the neuroimaging analysis, we appreciate the reviewer's suggestion to use parametric modulators more comprehensively. To address this, we conducted two additional GLM analyses (GLM2 and GLM3 in the revision):

GLM2: We specified a model with self-relevance as an onset regressor and relative allocation as a parametric modulator to directly test for brain activity tracking the magnitude of relative allocation (detailed in revised Methods, Page 20, Line 706-709).

However, GLM2 testing the parametric effect of allocation did not yield significant results (detailed in revised Results, Page 12, Line 354-359), suggesting the activations reported in the originally reported GLM (GLM1 in the revision) may reflect the categorical effect of self-relevance rather than a linear effect of allocation amount. We have revised the manuscript to clarify and discuss this point (revised Discussion, Page 15, Line 459-463).

GLM3: We specified a simpler model with a single onset regressor across all trials (without condition-specific regressors) and relative performance as a parametric modulator to test a generic performance-tracking signal, as suggested (detailed in revised Method, Page 20, Line 709-712).

GLM3 yielded only weak activations in the dmPFC, but did not survive FWE correction. This suggested that a simple model of performance tracking is insufficient, and that the significant parametric effects in the originally reported GLM (GLM1 in the revision, which are modulated by self-relevance) are indeed more sensitive and theoretically meaningful (revised Results, Page 12, Line 366-371).

Furthermore, in the interest of clarity and focus, we have removed the group-level covariate analysis from the revised manuscript (as also suggested by Reviewer #3) because its inclusion was confusing and not central to our primary hypotheses. As the covariate included at the group level is independent of the parametric modulation effect, removing the covariate analysis does not influence the result of parametric modulation.

Manuscript changes:

We have incorporated descriptions of GLM2 and GLM3 into the Methods (revised Methods, Page 20, Line 706-712): “Furthermore, to assess neural markers associated with the magnitude of relative allocation, we specified a second GLM (GLM2) that included the onset regressors for self-relevance, with relative allocation entered as a parametric modulator for each onset regressor. A third GLM (GLM3) was constructed with a single onset regressor encompassing all trials and relative performance was included as a parametric modulator to examine brain activity related to a basic effect of performance independent of other factors.”

The Results section now reports their outcomes.

Revised Results (Page 12, Line 354-359): “Additionally, we tested the parametric effect of allocation by conducting an alternative GLM (see GLM2 in Methods) that modeled self-relevance as onset regressors with relative allocation entered as a parametric modulator. The contrast for the self-relevance onsets revealed brain activations similar to those in the initial GLM1, Table S5-2. However, we did not observe significant brain activity related to the parametric modulation of relative allocation. These results suggest that neural responses during allocation primarily reflect self-relevance rather than the graded effects of allocation, indicating that the self-allocation bias is represented in brain regions implicated in social decision-making (37,39,60)” (Page 12, Line 366-371): “In a simplified GLM that included only relative performance as a parametric modulator without condition regressors (see GLM3 in Methods), we did not find significant performance-related activations. Instead, the parametric effect of performance emerged when contribution levels were modeled as described in GLM1, i.e., with three onset regressors corresponding to the contribution levels and relative performance entered as a parametric modulator for each regressor.”

And the Discussion has been revised to interpret our neural findings in light of these new analyses, clarifying that they reflect self-relevance and context-dependent performance evaluation.

Revised Discussion (Page 15, Line 459-471): “Through additional control analyses, we gained finer-grained insights into these neural correlates. The absence of a parametric effect of allocation magnitude (GLM2) suggests that the medial prefrontal, insula and TPJ activations are linked to the cognitive state of making a self-referential decision, rather than to the specific output of that decision.

In contrast, the fact that performance-related activity in the medial and lateral prefrontal as well as and temporoparietal regions was only detectable in a model that accounted for contribution level (GLM1) and not in a generic model (GLM3), underscores that these regions are not merely tracking performance, but are computationally involved in integrating performance with the causal structure of the task to inform social decisions. While a simple GLM analysis aimed at identifying general neural correlates of relative performance failed to reveal significant brain activations, incorporating factorial regressors for self-relevance and contribution allowed us to better capture the underlying neural effects of trial-by-trial variation in relative performance.”

**#4-**The self-allocation bias reported here is related to self-serving bias. Please make a link to it.

***Response*:** We thank the reviewer for pointing out this important connection. The ‘self-allocation bias’, we describe, is indeed a specific and behaviorally measurable manifestation of the broader, well-established self-serving bias. The self-serving bias traditionally encompasses a tendency to attribute positive outcomes to internal factors and negative outcomes to external factors. Our study extends this concept from the domain of causal attribution (e.g., taking credit for success and denying blame for failure) and performance evaluation to the domain of resource distribution. We have now explicitly integrated this conceptual link throughout the manuscript (revised Introduction: Page 4, Line 104-112; revised Discussion: Page 14, Line 419-426).

Manuscript changes:

Revised Introduction (Page 4, Line 104-112): We now introduce the self-serving bias early on as a foundational concept that predicts self-interested behavior across domains, thereby motivating our investigation into its expression in performance-based allocations.

“A key driver of such deviations is the pervasive influence of self-serving biases (15,19,20). This well-established tendency for individuals to process information in a self-advantageous manner manifests not only in attribution (e.g., taking credit for success and denying blame for failure) but also in behavior, such as exerting less effort for others’ benefit (21). We propose that this general self-serving tendency should also powerfully extend to the domain of resource distribution, leading to a systematic ‘self-allocation bias’—where individuals overweight their own interests when dividing jointly earned rewards. This bias is expected to be modulated by self-relevance.”

Revised Discussion (Page 14, Line 419-426): We have strengthened the discussion to position our 'self-allocation bias' as a specific, behaviorally measurable type of self-serving bias, thereby extending the theoretical scope of this classic bias from purely cognitive attributions to consequential social decision-making.

“Our findings revealed that the self-allocation bias appeared not only as a baseline preference for self-benefitting allocation distribution but also as a reduced reliance on own actual performance. In other words, individuals prioritized personal advantage over objective performance information to distribute resources in a self-serving manner. Therefore, the self-allocation bias examined in the present study can be understood as a specific and behaviorally measurable manifestation of the broader self-serving bias, extending it from attributional judgements to concrete decisions of resource distribution.”

**#5-** In Experiment 3, the authors report that the allocation bias is driven more by a tendency to allocate additional resources to oneself than by a tendency to overestimate one's own performance. First, it would be helpful to report the full results of this experiment. Did participants also show any evidence of overestimating their performance? Additionally, in the other experiments, participants were shown their actual performance—was this also the case in Experiment 3? If so, given that there was no ambiguity about performance, was it even possible to detect any effect of an inflated self-performance estimate?

***Response*:** We thank Reviewer #1 for this insightful question, which allows us to clarify a key and nuanced aspect of our experimental design. First, we have now reported the full results of Experiment 3 in the SI, replicating the main behavioral findings (revised supplementary Fig S4, S5, and Table S7).

The reviewer is absolutely correct that in all experiments, including Exp. 3, participants were presented with matched, objective unambiguous, and matched performance data, leaving no room for a literal, numerical overestimation of objective performance.

However, the bias we aimed to capture is more subtle and implicit. It operates at the level of subjective perception and subjective importance that individuals assign to their own objective performance versus another’s identical objective performance when making allocation decisions. That is, how much influence does one's subjective weighing of own performance have on one's allocation decision? In the first two experiments, the difference in slopes of relative performance indicated that even when performances were objectively matched and clearly displayed, individuals processed and utilized that information in a self-serving manner.

Exp. 3 was not designed to detect an "inflated self-performance estimate" in terms of number perception, but to further dissect the source of this biased weighting. Does it stem primarily from an overvaluation of one's own performance as a source of information ("My score of '5' correct answers is more important than your '5'"), or from a preference for oneself as a recipient of rewards ("I want to give more to me, regardless of the performances")? Our results robustly pointed to the latter mechanism.

In summary, by holding objective performance constant and matched, our design aimed to isolate the self-serving bias in the decision-weighting process itself, rather than in the initial perception of performance. These points were clarified in the revision (revised Results: Page 9, Line 284-292; Revised Discussion, Page 14, Line 426-429).

Manuscript changes:

Revised Results (Page 9, Line 284-292): “In our current design, self-relevance is tied to both who performs and who receives the allocations. Consequently, the self-allocation bias cannot be disentangled as arising from the source of performance or from the recipient of allocations, since both always refer to the self in the self-relevant condition (self-performance for self-allocation) and to another person in the self-irrelevant condition. To further extend our understanding of the self-allocation bias, we conducted an additional Experiment 3 (N = 62) to specifically examine the separate impacts of the “allocation recipient” and “performance source” that led to the outcomes to-be-allocated. For reasons of brevity, we present task details, methodology, and results in the SI (Fig S4, S5, Table S7). In brief, we structured self-relevance into two distinct factors: performance source and allocation recipient.”

Revised Discussion (Page 14, Line 426-429): “Evidence from Experiment 3 further supports this interpretation. By disentangling two potential mechanisms, it showed that the observed bias stemmed primarily from a preference for oneself as the recipient of rewards rather than from an inflated evaluation of own performance.”

**#6-** "Thus, the activities of both mPFC and TPJ suggest an integration of self-referential processing and mentalizing networks for self-other allocation decisions." (Line 438).  Can the authors clarify how their task is related to mentalising? Just because they found activation in TPJ/mPFC does not mean that their task requires mentalising. If the current task has an element of mentalising in it, the authors should mention that and explain why. Linking a process to mentalising just because it recruits the brain regions which light up during mentalising is reverse inference I am afraid.

***Response*:** We thank the reviewer for cautioning us against reverse inference. We apologize for the use of reverse inference in the original manuscript and completely agree that mere TPJ/mPFC activity does not demonstrate mentalizing or self-referential processing. We have thoroughly revised our interpretation to be driven by task-based reasoning.

Our task requires participants, as the allocator, to evaluate the performance and make a monetary allocation decision between themselves and another person. This design engages at least two core social cognitive processes: i) self-referential processing & value computation (evaluating performance in relation to their own payoff; and ii) other-benefit consideration, perspective-taking. To make a (even minimally) socially acceptable or strategic allocation, the allocator must also consider the other person's expectation based on their performance. This requires going beyond a purely selfish calculation to represent the other's perspective or expectation, a process that has been linked to the TPJ in the previous work (Hutcherson et al., 2015; Morishima et al., 2012; Soutschek et al., 2016; Speitel et al., 2019).

Therefore, we theoretically predicted the involvement of these regions based on the task demands. The observed activations in mPFC and TPJ are consistent with—and provide neural evidence for—the engagement of these processes during the task. We have rewritten all relevant sections of the manuscript to reflect this task-, theory-driven logic and to avoid any language suggestive of reverse inference

Manuscript changes:

Revised Introduction (Page 5, Line 148-161): “Crucially, we investigated the neural underpinnings of such a self-allocation bias during performance-based social allocation. Making self-relevant allocation decisions requires the computation of self-relevant value and the consideration of the other person’s claim. Midline regions of the brain, such as the medial prefrontal cortex (mPFC), the anterior cingulate cortex (ACC), and the posterior cingulate cortex (PCC), have been shown implicated in processing self-referential information and subjective value (36–43). We therefore expected these regions to be engaged when allocating joint outcomes for the self as compared to allocating joint outcomes for others. The anterior insula, which reacts to social norm violation and inequity (44–46), might be involved in the trade-off between self-interest and fairness when allocating joint outcomes in a self-serving way. Furthermore, the need to consider the other individual’s perspective in a cooperative setting led us to expect engagement of brain regions typically associated with evaluating others and integrating self-other information during social decision-making, such as the mPFC, bilateral temporoparietal junction (TPJ), superior temporal sulcus (STS), and temporal pole (TP) (43,47–51).”

Revised Discussion (Page 14, Line 444-453): “Both the mPFC and TPJ have been found to be associated with decision-making in social economic games (61,71) and with evaluating or comparing self to others (72–74). To make strategic allocation between self and others, individuals must going beyond a purely selfish calculation to also consider the other person's expectation based on their performance, which has been linked to the TPJ in the previous work.(64,75–77) Thus, the joint engagement of these regions in the current task is consistent with the idea that the self-referential processing and other-benefit consideration were both recruited during self-other arbitration in resource allocation. The observed activation in the insula may further indicate the motivation of self-benefitting allocation, consistent with its role in self-serving attribution (78,79).”; (Page 16, Line 487-494): “The TPJ appears to be involved in multiple aspects of the performance-based allocations between partners. Prior research has shown that the TPJ is activated during cooperative interactions (86), particularly during task success in group cooperation (87) and during evaluations of how contribution influences fairness considerations (88). As a key component during social decision-making (18,64,89,90), the TPJ performs a general integrative function of processing information about the performance of self and others along with information about the contribution to the group’s success, to then transform the information into subsequent social allocations.”; (Page 16, Line 527-531): “Neural evidence highlights the involvement of self-referential processing and social decision-making networks, mainly in the midline regions and the TPJ. Our findings provide novel insights for understanding how humans make performance-based allocation decisions during social cooperation by specifying how self-related biases and individual contributions jointly shape neural activity in prefrontal and temporal regions.”

**Minor comments:**
-Line 81: "task criteria impacted on social allocation…". There is an extra "on", it should be task criteria impacted social allocation…

***Response*:** Thank you, and we have corrected the sentence accordingly (Page 3, Line 90).

-Line 100:  "found that individuals allocated consistently higher resources to their future selves compared to allocations made for others." This sentence is convoluted. Does consistency refer to resources or allocation. If the latter, it should be individuals consistently allocated…, otherwise it should be clarified.

***Response*:** Thank you for pointing out this misleading point. As this paper showed a difference in allocations to future self and others even after accounting for some relevant factors, the consistency refers to the effect of target on allocation. We have corrected the phrase to “…individuals consistently allocated higher resources…” in the revised manuscript (Page 4, Line 115-116).

-Line 157 "reflects the structure knowledge of the environment". Was it meant to be "reflects knowledge of the structure of the environment"? or "reflects the structural knowledge of the environment"? please clarify.

***Response*:** Thanks and we have revised the sentence as follows for clarity: (Page 5, Line 163) “…reflects knowledge about the structure of the environment.”

-It will save reviewers quite a bit of time if the figures are placed in the text where they are relevant followed by their captions. Putting the text, the figures and the captions each in a different place does not help anyone.

-In addition, the quality of the figures is extremely low. Figure 1 is illegible. Please replace them.

***Response*:** Thank you. All figures have been re-created with high resolution and are now placed close to their citation in the text.

-Line 260: "we examined in our third hypothesis". It should be "we examined our third.."

***Response*:** Thank you, and we have corrected the sentence accordingly (Page 9, Line 262).

-Line 276: "So far, our design does not allow us to dissociate the self-relevance regarding performance source or allocation recipients since it consistently pertained to whose performance and allocations were considered."  This sentence is incomprehensible. Please rewrite.

***Response:*** Thank you, and this sentence has been completely rewritten for clarity: (Page 9, Line 284-288): “In our current design, self-relevance is tied to both who performs and who receives the allocations. Consequently, the self-allocation bias cannot be disentangled as arising from the source of performance or from the recipient of allocations, since both always refer to the self in the self-relevant condition (self-performance for self-allocation) and to another person in the self-irrelevant condition.”

-Line 349 "Second, we tested whether activity varied according to individual differences in the self-allocation bias in the interaction between self-relevance and contribution levels. The result showed that the behavioral self-allocation bias was linked to the posterior..". I am not sure what it is that the authors tested here. Did you test the effect of the interaction or the effect of allocation bias? Please clarify.

***Response:*** We thank the reviewer for pointing out this lack of clarity. This sentence referred to an exploratory analysis where we tested whether individual differences in the behavioral self-allocation bias correlated with the neural interaction effect between self-relevance and contribution.

However, upon further reflection (and in line with our response to Reviewer #3's point #3), we have decided to remove this exploratory analysis from the revised manuscript. This decision was made to sharpen the focus of the paper on our primary, hypothesis-driven findings and to avoid any potential confusion. The sentence in question has been deleted.

Line 437: and comparing self with others others. There seems to be an extra others.

***Response*:** Thank you, and we have removed the extra "others".

# **Reviewer #2**

Korn et al. used a novel effort-based reward allocation task to study how self-allocation bias affects reward allocation in cooperative tasks and investigated the neural mechanisms underlying this process. The task itself is novel, and the behavioral results are interesting and compelling. The neural analyses use conservation statistical thresholds, which is great. However, there are major issues that make the manuscript unpublishable in its correct form in a high impact journal like PLOS Biology. First, and more importantly, the authors are making claims about human social behavior (and their neural processes) using a new, previously untested task design with very small sample sizes, and these effects were not preregistered (at least that was not stated in the paper). This is not acceptable scientific practice for social neuroscience in 2025. The neural effects also appear to be very exploratory though the introduction is written as if only specific hypotheses were being tested. An appropriate middle ground to allay concerns around reproducibility and exploratory analyses is to address the comments below and perform an independent behavioral replication in a much larger sample (see comment 1-2).

***Overall response to Reviewer #2’s comment:*** We thank the reviewer for the thoughtful comments and the acknowledgment of our task's novelty and compelling behavioral results. In response to the reviewer’s comments, we clarified the study’s non-preregistered status, evaluated the statistical power, and implemented Bayesian hierarchical approaches to ensure the robustness of our findings. We also detailed the believability manipulation in the task and conducted a rigorous control analysis. We performed additional ROI analyses and further refined the interpretation of the fMRI results to enhance the accuracy and clarity of our conclusions. Below, we provide a point-by-point response.

**Major comments**
**#1)** I understand that the authors collected an initial behavioral sample and then re-ran the task for fMRI data collection. Since the second behavioral data set was a presumed replication, did the authors preregister these effects? If so, please state, and if not, please explain. Similarly, can the authors state whether any of the neural hypotheses were preregistered?

**#2)** The sample sizes in this study are very small, which is especially concerning with a new social paradigm. Further, the practice of collecting a very small behavioral sample in study 1, and also in study 2, and then combining these samples in the main text seems misleading. To go forward with publishing the behavioral and neural results of a new economic task, the authors should show a pre-registered replication in a much larger sample of least N = 200. This would add a level of confidence in the reliability of the task and the authors claims.

***Response*:** We appreciate the reviewer's concern regarding reproducibility. As also noted by the Editor, the robustness and reliability of our key behavioral effects are supported by consistent replication across three independent experiments, as demonstrated not only in the combined analysis of Experiments 1 and 2 presented in the main text, but also in the individual results of Experiment 1, Experiment 2 (revised supplementary Fig S1, S2, Table S1-S4), and Experiment 3 (revised supplementary Fig S5, Table S7).

While we fully acknowledge the value of large-scale, preregistered replications for novel paradigms, conducting a new study with a sample size of N=200 exceeds the practical scope of this revision. Instead, we conducted analytic power calculation of the major tests in the experiments to ensure that our current datasets provided sufficient power and sensitivity to detect the observed effects. The analytic calculation of “minimum detectable effect” (MDE) has been used in power analysis (Bloom, 1995; Djimeu & Houndolo, 2016). In our main analyses with the fixed effects of self-relevance or contribution, the model estimates exceeded the MDEs at 80% power in all three experiments, which indicates that our dataset has sufficient sensitivity to detect the observed effects of this magnitude.

Additionally, in the revised manuscript, we have implemented hierarchical models, specifically Bayesian multilevel models, instead of ANOVAs as our primary statistical approach (Page 18, Line 622-627). This method stabilizes parameter estimation and improves effective power in small samples or unbalanced data, which shows significant advantages over traditional frequentist approaches that are highly sensitive to sample size (Baldwin & Fellingham, 2013; Gelman et al., 2013; McNeish, 2016).

We have acknowledged this as a limitation and explicitly stated in the revised Methods section (Page 18, Line 638-640) that the study was not preregistered. To enhance transparency, we have further clarified that the research hypotheses were formulated a priori, prior to data collection, though they were not formally preregistered

Manuscript changes:

Revised Methods (Page 18, Line 622-627): “As the relative allocation has zero-one-inflated beta distribution, we implemented Bayesian generalized non-linear multilevel models to test our hypotheses by using the “brms” package in R Studio with the family of zero_one_inflated_beta(). This method stabilizes parameter estimation and improves effective power in small samples or unbalanced data, which shows significant advantages over traditional mixed models that are highly sensitive to sample size (98–100).”; (Page 18, Line 638-640): “In the interest of transparency, we note that the study was not preregistered. However, the research hypotheses were developed prior to data collection but were not formally pre-registered.”

**#3)** Were the partners in the allocation task synthetic agents? If so, did participants think these were real partners? Did the authors check for believability of this manipulation?

***Response*:** We thank the reviewer for raising this critical methodological point. We would like to clarify and provide new evidence to address this concern (revised Results section: Page 5, Line 180-185; revised Methods: Page 17, Line 555-561; Page 19, Line 671-677).

Believability Manipulation: The task performances were indeed pre-programmed by design to ensure matched performance across trials between the self and partner in the self-relevant condition and between the two agents in the self-irrelevant condition. However, to establish a credible social context, four participants were scheduled to arrive simultaneously and were tested in pairs. Each participant was led to believe that their task partner was one of the other individuals with whom they had arrived, and that performances were based on a random subset of trials from their earlier task performance. Additionally, participants’ compensation was also determined by a random selection of trials in the allocation task, and this procedure was explicitly explained to them during the practice session. Together, these procedures enhanced the believability of the social context.

Post-experiment checks and control analysis: Crucially, we asked participants to report whether their decisions were influenced by suspicions regarding the reality of the partners or their performance feedback. A high percentage of participants across all experiments (86.5% on average) reported that their decisions were not influenced (Exp1: 31/36; Exp2: 28/35; Exp3: 56/62).

To quantitatively address any potential confound, we performed a rigorous control analysis: we excluded all participants who expressed doubts about the reality of the partners and reported that this belief influenced their decisions (Exp1: 5 excluded, final N=31; Exp2: 7 excluded, final N=28; Exp3: 6 excluded, final N=56). All our key behavioral results remained statistically unchanged in these purified samples (revised Figure S5, S6).

Manuscript changes:

Revised Results (Page 5, Line 180-185): “To ensure a credible social context, four participants were scheduled simultaneously and tested in pairs, under the belief that their partner was one of the other participants and that task performance depended on a random subset of earlier trials in the performance task. A control analysis confirmed that the results remained robust in a sub-sample when excluding participants who were influenced by doubts about task manipulation (see *Methods* and *Supporting information (SI)* **Fig S6, S7**).”

Revised Methods (Page 17, Line 555-561): “To establish a credible social context, four participants were scheduled to arrive simultaneously and were tested in pairs. Each participant was led to believe that their task partner was one of the other individuals with whom they had arrived, and that performances were based on a random subset of trials from their earlier task performance, independent of their overall performance. Additionally, part of each participant’s compensation was also determined by a random selection of trials in the allocation task, and this procedure was explicitly explained to them during the practice session.”; (Page 19, Line 671-677): “Post-experiment check revealed that a high percentage of participants across all experiments (86.5% on average) reported that their decisions were not influenced by whether the other players in the task were real or fictitious. We performed a rigorous control analysis to exclude participants who expressed doubts about the reality of the partners and reported that this belief influenced their decisions (Experiment1: 5 excluded; Experiment 2: 7 excluded: additional Experiment 3: 6 excluded). All our key behavioral results remained statistically unchanged in these purified samples (**Fig S6, S7**).”

**#4)** I appreciate that the authors are trying to convey the task frames to readers to explain the task, but the figure itself fails to explain what the actual task was. What were the questions/prompts that people were attempting to answer in the scanner? Also, not sure if this is just an English translation issue, but to state "You corrected 3" is very confusing. Were the participants correcting errors as part of the task? If the authors instead mean to communicate how many answers were correct on a performance task, please include the actual language that was relayed to participants.

***Response*:** We agree completely and apologize for the lack of clarity in the original figure. In the revision (Page 6), we have completely redesigned Figure 1 to make it more intuitive. The new figure now includes a clearer explanation of the condition manipulations and provides detailed descriptions of the task procedures. In addition, the figure caption has been revised to explicitly state the exact wording used, such as clarifying that "M correct 3" means "Player M had 3 correct answers” and to specify the exact responses participants were required to make. We are confident that the revised figure now provides a crystal-clear depiction of the task.

**#5)** The neural results of this paper appear highly exploratory. Although the authors explain hypothesized involvement of self-referential/mentalizing brain regions, such as ACC, mPFC, dmPFC, and TPJ, the authors do not motivate the involvement of other regions known to contribute to social decision-making, such as ventral striatum and hippocampus, although these regions are described in the results. If the authors hypotheses were only related to regions described in the introduction, why did the authors conduct a whole-brain analysis rather than ROI-based analyses? I think the main issue is that the introduction is written as if the authors are tested very specific hypotheses, but the results are written as if the analyses conducted were exploratory. For instance, looking for individual differences in self-allocation bias in the hippocampus and ventral striatum seems very post-hoc and exploratory since neither of these subcortical regions, which of course have their own specializing functions, are theoretically motivated. So, either the introduction needs to state that there were some hypothesized effects, but the actually conducted analyses were exploratory, or the authors should state in the results sections which results and analyses were directly related to the main study hypotheses, and which were exploratory/serendipitous findings.

***Response*:** This is an extremely important point. We thank the reviewer for this comment, which motivated us to refine our reporting strategy. We indeed had specific regions of interest (as mentioned in the original introduction), but we reported results from whole-brain analysis. Our original intention was to use the robust, conservative whole-brain analysis as the primary method to test our hypotheses regarding a priori regions, while also being open to exploratory findings. This choice is made because the mPFC is a relative large and functionally heterogeneous regions and the TPJ is not strictly anatomically defined (Vega et al., 2016; Klein-Flügge et al., 2022; Schurz et al., 2014; Mars et al., 2012). Therefore, we did not specify precise locations or subregions as a priori. We acknowledge that the manuscript did not clearly communicate this strategy. In response, we have taken the following steps:

1) Clarified analysis strategy: We have revised the Introduction (Page 5, Line 169-171) and Methods (Page 20, Line 719-724) to explicitly state that we employed whole-brain analysis as our primary, conservative approach to test our hypotheses regarding the broad, functionally heterogeneous regions of mPFC and TPJ, while also being open to exploratory findings in other regions.

2) Conducted complementary ROI analyses: To directly address the reviewer's question and bolster our key findings, we have now conducted formal ROI analyses for our two main contrasts (self-relevance and performance modulation). We defined anatomical ROIs for mPFC, insula, and TPJ, and a functional ROI for dmPFC based on prior literature (Wittmann et al., 2016). We are pleased to confirm that the activations reported in our whole-brain analysis remain statistically significant within these pre-defined ROIs. The full results of these analyses have been detailed in the Response Table R1 but not in the main text, to maintain a clean and consistent reporting standard throughout the manuscript.

3) Removed exploratory covariate analysis: The covariate analysis was an exploratory attempt that did not hold after the control analysis and was also not central to our main hypotheses. We have therefore removed the covariates analysis from the revised manuscript (also in response to Reviewer #3’s point #3). This decision sharpens the focus of our paper and eliminates the post-hoc appearance the reviewer rightly highlighted.

Finally, we have improved the manuscript organization and have clarified this reasoning to ensure that the analytical approach and its relation to our hypotheses are more transparently communicated.

Manuscript changes:

Revised Introduction (Page 5, Line 169-171): “We employed a conservative, whole-brain analysis as our primary approach to test these hypotheses concerning these broad, functionally heterogeneous regions.”

Revised Methods (Page 20, Line 719-724): “Since the mPFC is a relative large and functionally heterogeneous regions and the TPJ is not strictly anatomically defined (101–104), we employed robust and conservative whole-brain analyses as the primary method to test our priori hypotheses regarding these regions, while remaining open to exploratory findings in other brain areas. Through this approach, we aimed to provide a comprehensive understanding of the neural mechanisms underlying performance-based social allocation decisions.”

Response Table R1. ROI analyses for the two contrasts reported in the revised manuscript.

|  | Side | x | y | z | Cluster size | p(FWEc) | Peak *T/F* | |
| --- | --- | --- | --- | --- | --- | --- | --- | --- |
| **Onset: Self-relevant > Self-irrelevant** | | | | | |  | *T* |  |
| Medial Frontal Gyrus (mPFC) | L/R | 18 | 54 | 24 | 2145 | <0.0001 | 8.01 |  |
| Insula | R | 34 | 19 | -10 | 127 | 0.001 | 5.93 |  |
| Angular Gyrus (TPJ) | R | 44 | -56 | 32 | 150 | <0.0001 | 4.25 |  |
| **Parametric modulation: Contribution** | | | | | |  | *F* |  |
| dmPFC | L/R | 0 | 42 | 36 | 129 | <0.0001 | 16.35 |  |
| Angular Gyrus (TPJ) | R | 44 | -64 | 42 | 529 | <0.0001 | 18.86 |  |

*Note*: ROI masks of mPFC, insula and TPJ were derived from the anatomical labels of the medial frontal gyrus, insula and angular gyrus, respectively. The dmPFC ROI was defined as a 10-mm spherical mask centered at x = 2, y = 44, z = 36 (Wittmann et al., 2016).

**#6)**      If I understand correctly, the parametric modulation analyses for the self-relevant conditions (player 1) effects are associated with reduced functional engagement of TPJ and dMPFC regions, as shown in Fig. 5. Is reduced functional activation of these regions the correct interpretation? In other words, are these results directionally consistent with the authors hypotheses? I could be wrong as the interpretation may just depend on the direction of the slope for the parametric modulation effect, so please clarify how the negative beta coefficients should be correctly interpreted.

***Response*:** We appreciate the reviewer's request for clarification, as the interpretation of parametric modulation results is indeed crucial. The reviewer is correct that the sign of the beta coefficient indicates the direction of the modulation, not the overall level of activation. The beta coefficients indicate how neural activity in a region scales with the magnitude changes in the parametric modulators. Specifically, in our analysis, the parametric modulator reflected trial-by-trial variation in relative performance of player 1, and was modeled separately for three contribution levels: “player 1 only” (only player 1 contributed to the rewards), “both” (both players contributed) and “player 2 only” (only player 2 contributed) conditions. The interpretation can be outlined like this:

The Parametric Modulator: We modeled relative performance of Player 1 (P1). Thus, a higher value means P1 did better; a lower value means P2 did better.

The Pattern: We found that in regions including the dmPFC, right MTG, bilateral OFC and TPJ, the beta weight for this modulator was negative in "P1 only" trials, near zero in "both" trials, and positive in "P2 only" trials.

The Interpretation: This pattern reveals that these regions are most sensitive to the performance of the player who did not cause the group to succeed.

In "P1 only" trials (negative beta), brain activity increases as P1's performance decreases (i.e., as the non-contributing P2's performance increases). In "P2 only" trials (positive beta), brain activity increases as P1's performance increases (i.e., as the non-contributing P1's performance increases).

In summary, these regions do not simply track one player's performance. Instead, they appear to selectively track the performance of the "non-essential" player, whose performance is irrelevant for the group outcome but critically important for deciding on the reward allocation. This sophisticated, context-dependent computation is a central finding of our study. We have revised the Results and Discussion to present this interpretation more clearly (revised Results: Page 12-13, Line 379-386; revised Discussion: Page 15, Line 472-483).

Manuscript changes:

Revised Results (Page 12-13, Line 379-386): “Moreover, these regions showed a consistent activation pattern related to contribution levels such that the beta estimates were negative in the “player1 only” condition, near zero in the “both” condition and positive in the “player2 only” condition, indicating a graded increase in activations across these contribution levels. This pattern suggests that these regions appear to selectively track the performance of the player who did not cause the group to succeed. Taken together, these results illustrate the neural underpinnings of how contribution levels influence performance-based allocation and further highlight the involvement of prefrontal and TPJ regions during this decision-making process.”

Revised Discussion: Page 15, Line 472-483): “Specifically, we observed a consistent pattern in all regions that the beta coefficients were negative in the “player 1 only”, near zero in the “both” condition, and positive in the “player 2 only” condition. As the parametric modulator reflected trial-by-trial variation in relative performance of player 1, a higher value means player 1 did better; a lower value means player 2 did better. In the “player 1 only” condition, negative beta coefficients suggest that activity in these brain regions encodes information about player 2’s performance in a positive direction. Conversely, in the “player 2 only condition”, positive coefficients suggest a positive link between neural activity and player 1’s performance. This graded pattern suggests a contribution-dependent sensitivity to the "non-essential" player, whose performance is irrelevant for the group outcome but critically important for deciding on the reward allocation. When only one player alone determines the reward, allocation decisions still require evaluating the other player’s performance, which becomes more informative in these situations.”

# **Reviewer #3**

The paper presents results of an fMRI experiment on social preferences. In the study, the participants perform a simple number sorting task and then make a series of monetary allocations. In one condition ("self-relevant"), these allocations are made between themselves and another person; in another condition ("self-irrelevant"), the allocation is made between two other people. The participant can use the results of the number sorting task to make the decision; there are 3 additional conditions: in the "simple" condition, the total reward is based on their individual performance; in the "additive" condition, it is based on some threshold their joint performance must reach; in the "disjunctive" condition, only one person had to reach the threshold.
The study finds that people tend to favor their own outcomes (as described in a large body of literature) but also tend to overlook their own poor task outcomes during allocations if that benefits them; this effect is stronger in people with low social value orientation. The paper then finds various neural correlates of certain decision variables (self-relevant vs self-irrelevant allocations, variations in performance, individual differences in self-allocation bias).
Strong qualities of the paper:
(1) The main idea of combining the social allocation with Shapley value-style contribution is novel and interesting.
(2) The results are clearly presented and the overall flow is easy to follow.
(3) The neuroimaging methods are clearly described and overall at the standard of the field.

***Overall response to Reviewer #3’s comment:*** We thank the reviewer for the positive assessment of our study and constructive suggestions. In response, we reported the believability manipulation and conducted a control analysis to confirm the robustness of our findings. We refined our discussion of limitations including the use of simulated interactions and the lack of precise measurement for computational modeling. We also removed the covariate analyses and carefully checked and updated the TPJ anatomical labels. Below, we provide a point-by-point response.

**Potential issues:**
**#(1)** As far as I could understand (it is not clearly described in the methods), the task uses deception (there are no real money allocations that are made) and the conditions are set up to fit the design. This is a common and significant issue in fMRI work in social neuroscience, as it is impossible to guarantee that participants base their decisions on actual preferences and not on experimenter demand effects. I do not see how this could be mitigated; I see this is a significant flaw that needs to be at least discussed, but of course, it's up to the editor to decide whether this flaw is critical for the publication in the journal.

***Response*:** We thank the reviewer for raising this crucial methodological point, which allows us to clarify two key aspects of our design that mitigated this concern. These were clarified in the revision (revised Results section: Page 5, Line 180-185; revised Methods: Page 17, Line 555-561; Page 19, Line 671-677; revised Discussion: Page 16, Line 511-516).

**First**, regarding the authenticity of the social interaction, we acknowledge that task performances were pre-programmed by design to ensure matched performance across trials. However, we implemented several measures to create a credible social context:

**i)** Four participants were scheduled to arrive simultaneously and were tested in pairs. Each participant was led to believe that their task partner was one of the other individuals with whom they had arrived, and that performances were based on a random subset of trials from their earlier task performance, independent of their overall performance.

**ii)** We conducted post-experiment checks. A high percentage of participants (86.5% on average) reported that their decisions were not influenced by suspicions about the reality of the partners or their performance feedback (Exp1: 31/36; Exp2: 28/35; Exp3: 56/62).

Most importantly, we performed a control analysis by excluding participants who expressed doubts and reported that this influenced their decisions. All key behavioral results remained statistically unchanged in these purified samples (revised Figure S5, S6), confirming the robustness of our findings against potential belief artifacts.

**Second,** regarding the monetary incentives, we would like to clarify that participants were informed that their monetary compensation would consist of a base show-up fee plus additional earnings determined by their allocation decisions in a few randomly selected trials. Specifically, they were told that several trials would be randomly chosen at the end of the experiment, and the allocations they made in those trials would be paid out. This procedure was explicitly explained to them during the practice session. This incentive structure ensured that participants faced real financial consequences for their decisions, encouraging genuine engagement with the task.

Manuscript changes:

Revised Results (Page 5, Line 180-185): “To ensure a credible social context, four participants were scheduled simultaneously and tested in pairs, under the belief that their partner was one of the other participants and that task performance depended on a random subset of earlier trials in the performance task. A control analysis confirmed that the results remained robust in a sub-sample when excluding participants who were influenced by doubts about task manipulation (see *Methods* and *Supporting information (SI)* **Fig S6, S7**).”

Revised Methods (Page 17, Line 555-561): “To establish a credible social context, four participants were scheduled to arrive simultaneously and were tested in pairs. Each participant was led to believe that their task partner was one of the other individuals with whom they had arrived, and that performances were based on a random subset of trials from their earlier task performance, independent of their overall performance. Additionally, part of each participant’s compensation was also determined by a random selection of trials in the allocation task, and this procedure was explicitly explained to them during the practice session.”; (Page 19, Line 671-677): “Post-experiment check revealed that a high percentage of participants across all experiments (86.5% on average) reported that their decisions were not influenced by whether the other players in the task were real or fictitious. We performed a rigorous control analysis to exclude participants who expressed doubts about the reality of the partners and reported that this belief influenced their decisions (Experiment1: 5 excluded; Experiment 2: 7 excluded: additional Experiment 3: 6 excluded). All our key behavioral results remained statistically unchanged in these purified samples (**Fig S6, S7**).”

Revised Discussion (Page 16, Line 511-516): “Another limitation of the present study is the use of simulated stimuli rather than real interaction during the task. Although our task design eliminated the need for direct interaction which is common in social fMRI tasks due to practical constraints and the need for experimental control, such approach still reduces the ecological validity of social interaction (94). Ensuring participants’ engagement and belief in the simulated social context is crucial for capturing neural and behavioral signatures of social cognition processes (95).”

**#(2)** The paper mentions some theoretical constructs, such as social preference functions and Shapley values, but lacks a proper theoretical foundation. What are the hypotheses that are being tested based on? What are the decisions in each trial based on? There are many computational/utility models of similar decisions and it would greatly benefit the paper to set up even a simple model that would estimate the values of various choices on each trial.

***Response*:** We thank the reviewer for this excellent suggestion to deepen the theoretical foundation of our work. We agree that formal modeling can provide a more mechanistic account of the allocation decision process and strengthen the interpretation of our findings. Here, our aim was to establish and thoroughly characterize a novel self-allocation bias. So, our task was not designed for testing computational models. But we indeed made some initial attempts, which made us realize that many requirements for adequate computational modelling are not met here. The primary limitation is that our behavioral measures (the allocation/performance ratio) is bounded and was designed to be symmetrically distributed. This design, while excellent for isolating the bias via ANOVA/mixed-effects models, substantially constrains the trial-by-trial variability and limits the interpretability and robustness of the model fitting.

We thus decided not to apply potentially underpowered model fitting in the current study. However, we do value the potential insights provided by computational models. We have integrated a discussion of this computational perspective into the Discussion section (revised Discussion: Page 16, Line 507-511), framing it as a crucial and logical next step.

Manuscript changes:

Revised Results (Page 16, Line 507-511): “Applying computational models could offer a more insightful mechanistic account of the allocation decision process. However, normalizing allocation and performance as ratios have constrained data variability and robustness of model fitting in the present study. Future studies could develop a refined task design that allows for greater behavioral variability and precise parameter estimation.”

**#(3)** The paper mentions individual variability results in the neuroimaging section, which are presented as activation maps. Given that the paper explores the individual relationships between SVO and choices in Figures 2 and 3, it would make sense to also show some scatterplots relating these neural activations to these variables on the individual level.

***Response*:** We thank the reviewer for this suggestion. We agree that visualizing individual differences can be powerful. In response, we did create scatterplots to visualize these individual differences. The combined nature of the contrasts produced confusing patterns that did not clearly reflect meaningful associations. This is possibly due to that the covariate analysis in question combined two contrasts, related to the dummy coding of the three contribution levels, which makes the resulting scatterplots difficult to interpret. Moreover, when we subjected it to a more rigorous check, the findings failed to remain statistically significant.

This analysis was exploratory, not central to our priori hypotheses, and its results were not reliable. Therefore, to maintain a sharp focus on our core, robust findings and to ensure the overall clarity of the manuscript, we have made the decision to remove this exploratory covariate analysis entirely from the revised manuscript. We believe this step strengthens the paper, as it eliminates a non-robust finding and allows us to maintain a clear and focused narrative on our core, well-supported results. We are grateful for the reviewer's input, which prompted this important re-evaluation.

**#(4)**     The activations labeled TPJ seem to be quite dorsal for TPJ and could potentially be IPS/IPL instead? These often co-activate with the lateral OFC. It might make sense to double check the regions and borders here.

***Response*:** We thank the reviewer for this precise anatomical observation, which prompted us to conduct a thorough and multi-faceted verification of our cluster locations. We agree that anatomical precision is paramount. However, the anatomical definition of TPJ is still debated without a clear conclusion (Mars et al., 2012; Patel et al., 2019; Schurz et al., 2014, 2017).We have carefully checked the regions labeled TPJ (***region a*** from the original Figure 4A and ***region b*** from the original Figure 5A) with three independent methods:

1) Anatomical labeling (panel A): We used the anatomy toolbox atlas labeling from bspmVIEW toolbox which includes the labeling of IPL. By checking at the anatomical labels of the borders (*region a*: Z = 28-42; *region b*: Z = 20-50), both regions were reported as “R Angular gyrus” rather than “R Inferior Parietal Lobule”, which is a core constituent of the TPJ complex.

2) Neurosynth association test (panel B): We used the terms “TPJ” and “IPL” in Neurosynth to get the image files from the association tests and overlayed them to our activations. Our clusters showed clear overlap with the "TPJ" meta-analytic map but were distinct from the "IPL" map.

3) Connectivity-based parcellation (panel C): Findings from connectivity-based parcellation atlases and meta-analyses (Mars et al., 2012; Schurz et al., 2014, 2017) indicate that the TPJ can be divided into three subregions based on the structural and functional connectivity: anterior TPJ (TPJa), posterior TPJ (TPJp) and the dorsal part of IPL. Referencing established atlases (Mars et al., 2012; Schurz et al., 2014), the central portions of our clusters align with the TPJp subregion.

This convergent evidence supports our original classification of these activations as part of the TPJ. However, to honor the reviewer's concern and achieve anatomical precision, we have updated our manuscript to refer to these regions more specifically as the "angular gyrus within the TPJ complex" where appropriate, in all relevant text, figures, and tables. We are grateful for the suggestion, which has enhanced the anatomical accuracy of our report.

**#(5)**     The figure panels could use a bit more labeling beyond the captions, as it can be difficult to determine which brain map corresponds to which contrast.

***Response*:** Thanks. We have thoroughly improved all figures. Specifically, we have added direct labels to each panel within the figures themselves. The captions have been expanded to ensure they fully describe the figure without requiring the reader to cross-reference the main text excessively (revised Fig 4, 5; revised figure captions: Page 12,13).

**#(6)**     The main neuroimaging results seem to only report positive activations. Were there no negative ones (it seems so from the supplementary table)? This is a bit unexpected and maybe deserves some mention/discussion.

***Response*:** This is a valid point. Our initial focus was on positive activations related to our hypotheses of self-relevance (self-relevant > self-irrelevant). In response, we also looked at the reversed contrast (self-irrelevant > self-relevant) and did not observe significant activation. In the revised manuscript, we now briefly mention the absence of significant negative contrasts in the Results section (revised Results: Page 12, Line 351-352), and provide further discussion in the Discussion section (revised Discussion: Page 15, Line 456-459). This ensures full reporting and transparency of all results.

Manuscript changes:

Revised Results (Page 12, Line 351-352): “In contrast, we did not observe any significant activations for the reversed contrast (self-relevant < self-irrelevant).”

Revised Discussion (Page 15, Line 456-459): “The trade-off between self-interest and norm violation may elicit greater motivational and emotional salience in the self-relevant condition than in the self-irrelevant condition, which could potentially explain the absence of brain activations in the reversed contrast.”

**References:**

Baldwin, S. A., & Fellingham, G. W. (2013). Bayesian methods for the analysis of small sample multilevel data with a complex variance structure. *Psychological Methods*, *18*(2), 151–164. https://doi.org/10.1037/a0030642

Bloom, H. S. (1995). Minimum Detectable Effects: A Simple Way to Report the Statistical Power of Experimental Designs. *Evaluation Review*, *19*(5), 547–556. https://doi.org/10.1177/0193841X9501900504

Djimeu, E. W., & Houndolo, D.-G. (2016). Power calculation for causal inference in social science: Sample size and minimum detectable effect determination. *Journal of Development Effectiveness*, *8*(4), 508–527. https://doi.org/10.1080/19439342.2016.1244555

Gelman, A., Carlin, J. B., Stern, H. S., Dunson, D. B., Vehtari, A., & Rubin, D. B. (2013). *Bayesian Data Analysis* (3rd ed.). Chapman and Hall/CRC. https://doi.org/10.1201/b16018

Hutcherson, C. A., Bushong, B., & Rangel, A. (2015). A Neurocomputational Model of Altruistic Choice and Its Implications. *Neuron*, *87*(2), 451–462. https://doi.org/10.1016/j.neuron.2015.06.031

Klein-Flügge, M. C., Bongioanni, A., & Rushworth, M. F. S. (2022). Medial and orbital frontal cortex in decision-making and flexible behavior. *Neuron*, *0*(0). https://doi.org/10.1016/j.neuron.2022.05.022

Mars, R. B., Sallet, J., Schüffelgen, U., Jbabdi, S., Toni, I., & Rushworth, M. F. S. (2012). Connectivity-Based Subdivisions of the Human Right “Temporoparietal Junction Area”: Evidence for Different Areas Participating in Different Cortical Networks. *Cerebral Cortex*, *22*(8), 1894–1903. https://doi.org/10.1093/cercor/bhr268

McNeish, D. (2016). On Using Bayesian Methods to Address Small Sample Problems. *Structural Equation Modeling: A Multidisciplinary Journal*, *23*(5), 750–773. https://doi.org/10.1080/10705511.2016.1186549

Morishima, Y., Schunk, D., Bruhin, A., Ruff, C. C., & Fehr, E. (2012). Linking Brain Structure and Activation in Temporoparietal Junction to Explain the Neurobiology of Human Altruism. *Neuron*, *75*(1), 73–79. https://doi.org/10.1016/j.neuron.2012.05.021

Patel, G. H., Sestieri, C., & Corbetta, M. (2019). The evolution of the temporoparietal junction and posterior superior temporal sulcus. *Cortex*, *118*, 38–50. https://doi.org/10.1016/j.cortex.2019.01.026

Schurz, M., Radua, J., Aichhorn, M., Richlan, F., & Perner, J. (2014). Fractionating theory of mind: A meta-analysis of functional brain imaging studies. *Neuroscience & Biobehavioral Reviews*, *42*, 9–34. https://doi.org/10.1016/j.neubiorev.2014.01.009

Schurz, M., Tholen, M. G., Perner, J., Mars, R. B., & Sallet, J. (2017). Specifying the brain anatomy underlying temporo-parietal junction activations for theory of mind: A review using probabilistic atlases from different imaging modalities. *Human Brain Mapping*, *38*(9), 4788–4805. https://doi.org/10.1002/hbm.23675

Soutschek, A., Ruff, C. C., Strombach, T., Kalenscher, T., & Tobler, P. N. (2016). Brain stimulation reveals crucial role of overcoming self-centeredness in self-control. *Science Advances*, *2*(10), e1600992. https://doi.org/10.1126/sciadv.1600992

Speitel, C., Traut-Mattausch, E., & Jonas, E. (2019). Functions of the right DLPFC and right TPJ in proposers and responders in the ultimatum game. *Social Cognitive and Affective Neuroscience*, *14*(3), 263–270. https://doi.org/10.1093/scan/nsz005

Vega, A. de la, Chang, L. J., Banich, M. T., Wager, T. D., & Yarkoni, T. (2016). Large-Scale Meta-Analysis of Human Medial Frontal Cortex Reveals Tripartite Functional Organization. *Journal of Neuroscience*, *36*(24), 6553–6562. https://doi.org/10.1523/JNEUROSCI.4402-15.2016

Wittmann, M. K., Kolling, N., Faber, N. S., Scholl, J., Nelissen, N., & Rushworth, M. F. S. (2016). Self-Other Mergence in the Frontal Cortex during Cooperation and Competition. *Neuron*, *91*(2), 482–493. https://doi.org/10.1016/j.neuron.2016.06.022
