# Supplementary material for: Self-allocation bias in performance-based cooperative decisions is driven by self-interest rather than distorted performance encoding
Source: PLoS Biol. 2026 Mar 26;24(3):e3003694. doi: 10.1371/journal.pbio.3003694 (PMC13020808; doi:10.1371/journal.pbio.3003694)
Supplement: Attachment — Submitted filename: PBIOLOGY-D-25-01892R2_Rebuttal letter_final.docx [file pbio.3003694.s018.docx]

**Overall response to the editors and reviewers:**

We are grateful for the constructive re-evaluation of our revised manuscript and thoughtful feedback. We are delighted to learn that the reviewers found our previous revisions helpful. In this round, we have carefully addressed the remaining concerns regarding the interpretation of the fMRI results (Reviewer #1) and the theoretical foundations of the observed behavior (Reviewer #3).

We believe that reorganizing the neural results and integrating these points into the *Discussion* has strengthened the paper’s interpretation and clarified its contribution to the field. Please find below our point-by-point responses.

***Responses to reviewers***

# Reviewer #1: Thank you for addressing my comments. It appears that the GLMs using task variables as parametric modulators do not yield meaningful results. What the fMRI data instead show is that certain brain regions are more active when participants allocate money to themselves compared with allocating money to others. I think the authors should clarify in the Discussion what these results do and do not imply, as well as the limitations of such a broad finding. For example, the observed self-relevance effect could be driven by expected reward in the self-relevant condition rather than self-related processing per se. As the results are currently presented, it is not straightforward—at least for me—to understand their precise interpretation.

***Response***: We thank the reviewer for this important comment regarding the interpretation and scope of the fMRI findings. To address this comment, we have substantially revised both the *Results* organization and the *Discussion* to more precisely delineate what our neural findings do and do not imply.

First, we clarify that the main effect of self-relevance observed in mPFC, anterior insula, and TPJ reflects a change in the decision context rather than a direct neural representation of reward magnitude or allocation outcome. This interpretation is supported by the absence of any parametric modulation by allocation magnitude in GLM2, indicating that these regions do not track trial-by-trial variations in how much money participants allocated to themselves or others. We now state this boundary condition in the Results and Discussion.

Second, and critically, the contribution of the current neural results lies not in the categorical self-relevance effect alone, but in the dissociation between self-relevance and performance encoding revealed by the contribution-dependent parametric modulation in GLM1 (Fig. 4 vs. Fig. 5).

We showed that relative performance was encoded in prefrontal and TPJ regions only when contribution structure was explicitly modeled (GLM1), and not in a context-independent model (GLM3). Importantly, this performance-related activity was modulated by contribution structure, but notably independent of self-relevance. As the parametric modulator encodes the information of both players’ performance using relative performance of one player, this modulation cannot reflect identity- or self-specific processing. Instead, the sign of the coefficients reflects tracking of the performance of the non-essential contributor, whose performance is informative but not causally necessary for group success.

We now clarify that this finding may constrain the interpretation of the self-relevance effect: behavioral self-allocation biases may not arise from biased neural encodings of performance itself, but potentially from how the performance information is used in allocation decisions when the self is involved. This refines existing accounts that conflate self-related processing with reward expectation and highlights a possible multi-stage decision process in which self-relevance gates how otherwise impartial performance information is used.

We also acknowledge that the temporal resolution of fMRI does not allow us to temporally resolve the precise stage at which self-relevance exerts its influence on decision computation, and the objective performance presentation may limit the potential representation of biased performance evaluation. However, the present results already placed constraints on candidate mechanisms by showing that self-bias did not arise at the level of performance encoding. Future studies using methods with high spatio-temporal resolution (such as sEEG) may directly test how self-relevance gates or transforms these representations during allocation decisions. These clarifications are now included in the revised Results and Discussion sections.

# **Reviewer #3:** I appreciate the effort the authors put in their revision. Many aspects of the methods and results were clarified, and my comments were mostly addressed. I noticed that other reviewers were somewhat critical, and I believe that the lack of a proper theoretical foundation for the decisions is an issue that could be important for a high-impact journal such as PLOS Biology. As the authors noted, this could not be addressed with the current design, so I can't recommend acceptance without hesitation, but overall, this is an interesting and potentially impactful paper.

***Response***: We thank Reviewer #3 for highlighting the importance of a clear theoretical foundation for the decision processes examined in this study. We agree that this issue is important for a high-impact journal such as PLOS Biology*.*

To address this issue, we have revised the manuscript to clarify the theoretical contribution of our findings beyond the task design itself. Rather than proposing a new model of allocation preferences, our results provide constraints on how performance information is encoded and used during allocation decisions by dissociating performance-encoding and decisional components of self-serving behavior. Specifically, we show that relative performance is encoded in a contribution-structured but self-independent manner in prefrontal and temporoparietal regions. Importantly, self-relevance does not distort these performance encoding at the neural level. This refines existing theories of self-serving bias by showing that self-serving outcomes may not require self-specifically biased inputs, but can instead emerge at later stages of decision computation. Experiment 3 provides behavioral support for this idea by showing only self-bias on allocation target but not performance source. In this sense, the performance-based allocation task serves not merely as a new paradigm, but as a potential tool for dissociating representational and behavioral components of self-serving bias.

While more formal computational models could further specify how these representations are transformed into behavior, the current results already provide principled constraints on candidate mechanisms. We have now included this theoretical framing into the revised Discussion.
